# Supplementary material for: Factors influencing the participation of pregnant and lactating women in clinical trials: A mixed-methods systematic review
Source: PLoS Med. 2024 May 30;21(5):e1004405. doi: 10.1371/journal.pmed.1004405 (PMC11139290; doi:10.1371/journal.pmed.1004405)
Supplement: S2 Appendix — (DOCX) [file pmed.1004405.s002.docx]

S2. Appendix: Enhancing Transparency in Reporting the Synthesis of Qualitative Research: (ENTREQ) reporting checklist

| No | Item | Guide and description | Location where item reported |
| --- | --- | --- | --- |
| 1 | Aim | State the research question the synthesis addresses | Introduction |
| 2 | Synthesis  methodology | Identify the synthesis methodology or theoretical framework which underpins the synthesis, and describe the rationale for choice of methodology (e.g., meta-ethnography, thematic synthesis, critical interpretive synthesis, grounded theory synthesis, realist synthesis, meta-aggregation, meta-study, framework synthesis). | Methods – Data analysis, and synthesis |
| 3 | Approach to  searching | Indicate whether the search was pre-planned (comprehensive search strategies to seek all available studies) or iterative (to  seek all available concepts until they theoretical saturation is achieved). | Methods – Search methods for identification of relevant studies |
| 4 | Inclusion criteria | Specify the inclusion/exclusion criteria (e.g., in terms of population, language, year limits, type of publication, study type) | Methods – Type of studies and Topic of interest |
| 5 | Data sources | Describe the information sources used (e.g., electronic databases (MEDLINE, EMBASE, CINAHL, psycINFO, Econlit), grey literature databases (digital thesis, policy reports), relevant organisational websites, experts, information specialists, generic web searches (Google Scholar) hand searching, reference lists) and when the searches conducted; provide the rationale for using  the data sources | Methods – Search methods for identification of relevant studies |
| 6 | Electronic Search strategy | Describe the literature search (e.g., provide electronic search strategies with population terms, clinical or health topic terms, experiential or social phenomena related terms, filters for qualitative research, and search limits) | Methods – Appendix S3 |
| 7 | Study screening  methods | Describe the process of study screening and sifting (e.g., title, abstract and full text review, number of independent reviewers who screened studies). | Methods – Selection of studies |
| 8 | Study characteristics | Present the characteristics of the included studies (e.g., year of publication, country, population, number of participants, data collection, methodology, analysis, research questions). | Results and Appendix S6 |
| 9 | Study selection  results | Identify the number of studies screened and provide reasons for study exclusion (egg, for comprehensive searching, provide numbers of studies screened and reasons for exclusion indicated in a figure/flowchart; for iterative searching describe reasons  for study exclusion and inclusion based on modifications to the research question and/or contribution to theory development). | Results and Figure 1. PRISMA flowchart |
| 10 | Rationale for  appraisal | Describe the rationale and approach used to appraise the included studies or selected findings (e.g., assessment of conduct (validity and robustness), assessment of reporting (transparency), assessment of content and utility of the findings). | Methods – Data extraction and assessing methodological limitations |
| 11 | Appraisal items | State the tools, frameworks and criteria used to appraise the studies or selected findings (e.g., Existing tools: CASP, QARI, COREQ, Mays and Pope [25]; reviewer developed tools; describe the domains assessed: research team, study design, data analysis and interpretations, reporting). | Methods – Data extraction and assessing methodological limitations |
| 12 | Appraisal process | Indicate whether the appraisal was conducted independently by more than one reviewer and if consensus was required | Methods – Data extraction and assessing methodological limitations |
| 13 | Appraisal results | Present results of the quality assessment and indicate which articles, if any, were weighted/excluded based on the assessment and give the rationale. | Appendix S7 |
| 14 | Data extraction | Indicate which sections of the primary studies were analysed and how were the data extracted from the primary studies? (e.g., all text under the headings “results /conclusions” were extracted electronically and entered into a computer software). | Methods – Data extraction and assessing methodological limitations |
| 15 | Software | State the computer software used if any | Methods – Selection of studies |
| 16 | Number of  reviewers | Identify who was involved in coding and analysis. | Methods – Selection of studies , Data extraction and assessing methodological limitations, Data analysis, and synthesis |
| 17 | Coding | Describe the process for coding of data (e.g., line by line coding to search for concepts) | Methods – Data analysis, and synthesis |
| 18 | Study comparison | Describe how were comparisons made within and across studies (e.g., subsequent studies were coded into pre-existing concepts, and new concepts were created when deemed necessary) | Methods – Data analysis, and synthesis |
| 19 | Derivation of  themes | Explain whether the process of deriving the themes or constructs was inductive or deductive. | Methods – Data analysis, and synthesis |
| 20 | Quotations | Provide quotations from the primary studies to illustrate themes/constructs and identify whether the quotations were participant quotations of the author’s interpretation. | Quotations not provided as this is qualitative evidence synthesis |
| 21 | Synthesis output | Present rich, compelling, and useful results that go beyond a summary of the primary studies (e.g., new interpretation, models of evidence, conceptual models, analytical framework, development of a new theory or construct). | Results and discussion |
