# Supplementary material for: Factors influencing the participation of pregnant and lactating women in clinical trials: A mixed-methods systematic review
Source: PLoS Med. 2024 May 30;21(5):e1004405. doi: 10.1371/journal.pmed.1004405 (PMC11139290; doi:10.1371/journal.pmed.1004405)
Supplement: S3 Appendix — (DOCX) [file pmed.1004405.s003.docx]

S3. Appendix: Search Strategies

Medline

| # | Searches |
| --- | --- |
| 1 | (pregnant or pregnancy or lactating or lactation or breastfeed* or breast feed*).ti,kf. |
| 2 | pregnant women/ or pregnancy/ or breast feeding/ or lactation/ |
| 3 | 1 or 2 |
| 4 | ((inclu* or exclu* or participat* or recruit* or enrol* or involvement or involving or eligib* or decision or barrier* or facilitat* or ethic* or consent or acceptability or vulnerab* or engag* or regulat* or ethic*) adj6 (pregnan* or lactating or lactation or breastfeed* or breast feed*) adj6 (trials or research or clinical studies)).ab. |
| 5 | clinical studies as topic/ or exp clinical trials as topic/ or biomedical research/ or research subjects/ |
| 6 | ((drug or drugs or pharmaceutical* or vaccine* or medication or medications) adj2 (development or approval or regulation or evaluation)).ti,kf,hw. |
| 7 | ((inclu* or exclu* or participat* or recruit* or enrol* or involvement or involving or eligib* or decision or barrier* or facilitat* or ethic* or consent or acceptability or vulnerab* or engag* or regulat* or ethic*) and (pregnan* or lactating or lactation or breastfeed* or breast feed* or women or gender) and (trials or trial or research or clinical studies)).ti,kf. |
| 8 | 5 or 6 |
| 9 | health knowledge attitudes practice/ |
| 10 | motivation/ |
| 11 | patient participation/ |
| 12 | patient selection/ |
| 13 | decision making/ |
| 14 | eligibility determination/ |
| 15 | acceptability/ |
| 16 | informed consent/ |
| 17 | risk factors/ |
| 18 | attitude to health/ |
| 19 | stakeholder participation/ |
| 20 | risk assessment/ |
| 21 | trust/ |
| 22 | ethics.fs. |
| 23 | ethics committees research/ |
| 24 | research design/ |
| 25 | 9 or 10 or 11 or 12 or 13 or 14 or 15 or 16 or 17 or 18 or 19 or 20 or 21 or 22 or 23 or 24 |
| 26 | (regulat* or challenge* or eligib* or decision or barrier* or facilitat* or ethic* or moral* or consent or acceptability or vulnerab* or willing* or determinant* or benefit* or factor or factors or qualitative or altruis* or opportunit* or represent* or underrepresent* or risk or risks or unfair or fair or fairness or "scientifically complex" or stakeholder* or concerns or "focus group*" or interview* or recommend* or incentive*).mp. |
| 27 | (perception* or perspective* or opinion* or view or views or viewpoint* or reasons or reason or attitude* or experience*).mp. |
| 28 | (policy or policies or guideline* or model* or initiative* or implementation or "pharmaceutical compan*" or "pharmaceutical industr*" or "drug compan*" or "drug industr*" or "research agenda" or classif* or reclassif* or rule or rules or law or laws or legal or legislation or governance or guidance or safety or assessment or "research design" or "study design" or "research subjects").mp. |
| 29 | ("presumptive exclusion" or "unfair exclusion").mp. |
| 30 | ((participat* or inclu* or recruit* or enrol* or involv* or engag*) adj5 (pregnan* or lactating or lactation or breastfeed* or "breast feed*")).mp. |
| 31 | 25 or 26 or 27 or 28 or 29 or 30 |
| 32 | (3 and 8) or 4 or 7 |
| 33 | 32 and 31 |
| 34 | (clinical trial or randomized controlled trial or controlled clinical trial or clinical trial protocol).pt. |
| 35 | 33 not 34 |

EMBASE

| # | Searches |
| --- | --- |
| 1 | exp *named groups by pregnancy/ or exp *pregnancy/ or exp *breast feeding/ or *lactation/ or (pregnant or pregnancy or lactating or lactation or breastfeed* or breast feed*).ti,kf. |
| 2 | ((inclu* or exclu* or participat* or recruit* or enrol* or involvement or involving or eligib* or decision or barrier* or facilitat* or ethic* or consent or acceptability or vulnerab* or engag* or regulat* or ethic*) adj6 (pregnan* or lactating or lactation or breastfeed* or breast feed*) adj6 (trials or research or clinical studies)).ab. |
| 3 | exp "clinical trial (topic)"/ or medical research/ or clinical research/ or drug research/ or research subject/ |
| 4 | ((drug or drugs or pharmaceutical* or vaccine* or medication or medications) adj2 (development or approval or regulation or evaluation)).ti,kf,hw. |
| 5 | ((inclu* or exclu* or participat* or recruit* or enrol* or involvement or involving or eligib* or decision or barrier* or facilitat* or ethic* or consent or acceptability or vulnerab* or engag* or regulat* or ethic*) and (pregnan* or lactating or lactation or breastfeed* or breast feed* or women or gender) and (trials or trial or research or clinical studies)).ti,kf. |
| 6 | 3 or 4 |
| 7 | attitude to health/ or attitude to pregnancy/ or patient attitude/ |
| 8 | exp motivation/ |
| 9 | patient participation/ |
| 10 | patient selection/ |
| 11 | decision making/ or patient decision making/ or exp medical decision making/ |
| 12 | eligibility/ or eligibility criteria/ or legal aspect/ |
| 13 | program acceptability/ |
| 14 | informed consent/ |
| 15 | exp risk/ |
| 16 | stakeholder engagement/ |
| 17 | trust/ |
| 18 | exp ethics/ |
| 19 | professional standard/ |
| 20 | methodology/ or experimental design/ or study design/ |
| 21 | 7 or 8 or 9 or 10 or 11 or 12 or 13 or 14 or 15 or 16 or 17 or 18 or 19 or 20 |
| 22 | (regulat* or challenge* or eligib* or decision or barrier* or facilitat* or ethic* or moral* or consent or acceptability or vulnerab* or willing* or determinant* or benefit* or factor or factors or qualitative or altruis* or opportunit* or represent* or underrepresent* or risk or risks or unfair or fair or fairness or "scientifically complex" or stakeholder* or concerns or "focus group*" or interview* or recommend* or incentive*).mp. |
| 23 | (perception* or perspective* or opinion* or view or views or viewpoint* or reasons or reason or attitude* or experience*).mp. |
| 24 | (policy or policies or guideline* or model* or initiative* or implementation or "pharmaceutical compan*" or "pharmaceutical industr*" or "drug compan*" or "drug industr*" or "research agenda" or classif* or reclassif* or rule or rules or law or laws or legal or legislation or governance or guidance or safety or assessment or "research design" or "study design" or "research subjects").mp. |
| 25 | ("presumptive exclusion" or "unfair exclusion").mp. |
| 26 | ((participat* or inclu* or recruit* or enrol* or involv* or engag*) adj5 (pregnan* or lactating or lactation or breastfeed* or "breast feed*")).mp. |
| 27 | 21 or 22 or 23 or 24 or 25 or 26 |
| 28 | clinical trial/ or randomized controlled trial/ or controlled clinical trial/ or clinical trial protocol/ |
| 29 | systematic review/ |
| 30 | (1 or 2) and 6 |
| 31 | 30 or 5 |
| 32 | 31 and 27 |
| 33 | 32 not (28 or 29) |
| 34 | limit 33 to conference abstract status |
| 35 | 33 not 34 |

Global Health

| # | Searches |
| --- | --- |
| 1 | pregnant women/ or pregnancy/ or exp breast feeding/ or lactation/ or human lactation/ |
| 2 | (pregnant or pregnancy or lactating or lactation or breastfeed* or breast feed*).ti,id. |
| 3 | 1 or 2 |
| 4 | clinical trials/ or randomized controlled trials/ or biomedical research/ or research/ |
| 5 | ((drug or drugs or pharmaceutical* or vaccine* or medication or medications) adj2 (development or approval or regulation or evaluation)).ti,hw. |
| 6 | 4 or 5 |
| 7 | ((inclu* or exclu* or participat* or recruit* or enrol* or involvement or involving or eligib* or decision or barrier* or facilitat* or ethic* or consent or acceptability or vulnerab* or engag* or regulat* or ethic*) adj6 (pregnan* or lactating or lactation or breastfeed* or breast feed*) adj6 (trials or research or clinical studies)).ab. |
| 8 | ((inclu* or exclu* or participat* or recruit* or enrol* or involvement or involving or eligib* or decision or barrier* or facilitat* or ethic* or consent or acceptability or vulnerab* or engag* or regulat* or ethic*) and (pregnan* or lactating or lactation or breastfeed* or breast feed* or women or gender) and (trials or trial or research or clinical studies)).ti,id. |
| 9 | 3 and 6 |
| 10 | motivation/ or incentives/ |
| 11 | exp participation/ |
| 12 | decision making/ |
| 13 | eligibility/ |
| 14 | acceptability/ |
| 15 | consent/ |
| 16 | stakeholders/ |
| 17 | ethics/ or bioethics/ or medical ethics/ |
| 18 | committees/ or advisory committees/ |
| 19 | (regulatory or regulations or decision or barrier* or facilitat* or ethic* or moral* or acceptability or vulnerab* or willing* or qualitative or altruis* or opportunit* or underrepresent* or unfair or fair or fairness or "scientifically complex" or stakeholder* or concerns or "focus group*" or incentive*).mp. |
| 20 | (challenge* or eligib* or consent or recommend*).ti,id,hw. |
| 21 | (represent* adj6 (pregnan* or breastfeed* or breast feed* or lactating or lactation or women or minorit* or gender*)).mp. [mp=abstract, title, original title, heading words, cabicodes words] |
| 22 | (perception* or perspective* or opinion* or views or viewpoint* or reasons or reason or experience or experiences).mp. |
| 23 | attitude*.ti,id,hw. |
| 24 | (policy or policies or initiative* or "pharmaceutical compan*" or "pharmaceutical industr*" or "drug compan*" or "drug industr*" or "research agenda" or rule or rules or law or laws or legal or legislation or governance or guidance or "research design" or "study design" or "research subjects").mp. |
| 25 | (guideline* or implementation or safety).ti,id,hw. |
| 26 | ("presumptive exclusion " or "unfair exclusion").mp. |
| 27 | ((participat* or inclusion or recruit or recruitment or enrol or enrolment or enrollment or involvement or involving or engage or engagement) adj4 (pregnan* or lactating or lactation or breastfeed* or "breast feed*")).mp. |
| 28 | 10 or 11 or 12 or 13 or 14 or 15 or 16 or 17 or 18 or 19 or 20 or 21 or 22 or 23 or 24 or 25 or 26 or 27 |
| 29 | 9 and 28 |
| 30 | 7 or 8 or 29 |

CINAHL

| # | Searches |
| --- | --- |
| 1 | ( (MH "Expectant Mothers") OR (MH pregnancy) OR (MH "pregnancy, high risk") OR (MH "breast feeding") OR (MH lactation) ) OR TI ( pregnant OR pregnancy OR lactating OR lactation OR breastfeed* OR "breast feed*" ) |
| 2 | AB ((inclu* or exclu* or participat* or recruit* or enrol* or involvement or involving or eligib* or decision or barrier* or facilitat* or ethic* or consent or acceptability or vulnerab* or engag* or regulat* or ethic*) N5 (pregnan* or lactating or lactation or breastfeed* or "breast feed*") N5 (trials or research or "clinical studies")) |
| 3 | (MH "clinical trials") OR (MH "randomized controlled trials") OR (MH "research, medical") OR (MH +"research subjects") OR (MH "clinical research") |
| 4 | TI ( (drug or drugs or pharmaceutical* or vaccine* or medication or medications) N1 (development or approval or regulation or evaluation) ) OR SU ( (drug or drugs or pharmaceutical* or vaccine* or medication or medications) N1 (development or approval or regulation or evaluation) ) |
| 5 | TI ( (inclu* or exclu* or participat* or recruit* or enrol* or involvement or involving or eligib* or decision or barrier* or facilitat* or ethic* or consent or acceptability or vulnerab* or engag* or regulat* or ethic*) and (pregnan* or lactating or lactation or breastfeed* or "breast feed*" or women or gender) and (trials or trial or research or "clinical studies") ) |
| 6 | S3 OR S4 |
| 7 | (S1 AND S6) OR S2 OR S5 |
| 8 | (MH "Attitude to Health") OR (MH "Health Knowledge") |
| 9 | (MH "Motivation") |
| 10 | (MH "Consumer Participation") |
| 11 | (MH "Patient Selection") OR (MH "Research subject recruitment") OR (MH "Research subject retention") |
| 12 | (MH "Decision Making") OR (MH "Decision Making, Patient") OR (MH "Decision Making, Clinical") OR (MH "Decision Making, Ethical") |
| 13 | (MH "Eligibility Determination") |
| 14 | (MH "Consent (Research)") OR (MH Consent) OR (MH "Patient Autonomy") |
| 15 | (MH "Risk Factors") |
| 16 | (MH "Attitude to Health") OR (MH "Attitude to Pregnancy") OR (MH "Attitude to Risk") OR (MH "Patient Attitudes") |
| 17 | (MH "Stakeholder Participation") |
| 18 | (MH "Risk Assessment") |
| 19 | (MH "Trust") |
| 20 | (MH "Ethics Committees") OR (MH "Institutional Review") |
| 21 | (MH "Study Design") OR (MH "Clinical Trials/MT/CL/ST/LJ") OR (MH "Randomized Controlled Trials/MT/LJ/ST/OG") |
| 22 | S8 OR S9 OR S10 OR S11 OR S12 OR S13 OR S14 OR S15 OR S16 OR S17 OR S18 OR S19 OR S20 OR S21 |
| 23 | regulat* or challenge* or eligib* or decision or barrier* or facilitat* or ethic* or moral* or consent or acceptability or vulnerab* or willing* or determinant* or benefit* or factor or factors or qualitative or altruis* or opportunit* or represent* or underrepresent* or risk or risks or unfair or fair or fairness or "scientifically complex" or stakeholder* or concerns or "focus group*" or interview* or recommend* or incentive* |
| 24 | perception* or perspective* or opinion* or view or views or viewpoint* or reasons or reason or attitude* or experience* |
| 25 | policy or policies or guideline* or model* or initiative* or implementation or "pharmaceutical compan*" or "pharmaceutical industr*" or "drug compan*" or "drug industr*" or "research agenda" or classif* or reclassif* or rule or rules or law or laws or legal or legislation or governance or guidance or safety or assessment or "research design" or "study design" or "research subjects" |
| 26 | "presumptive exclusion" or "unfair exclusion" |
| 27 | (participat* or inclu* or recruit* or enrol* or involv* or engag*) N4 (pregnan* or lactating or lactation or breastfeed* or "breast feed*") |
| 28 | S22 OR S23 OR S24 OR S25 OR S26 OR S27 |
| 29 | S7 AND S28 |
| 30 | PT ("clinical trial" OR "randomized controlled trial" OR "protocol") |
| 31 | PT "systematic review" |
| 32 | S29 NOT S30 |
| 33 | S32 NOT S31 |

SocIndex, Family & Society Studies Worldwide

| # | Searches |
| --- | --- |
| 1 | (inclu* or exclu* or participat* or recruit* or enrol* or involvement or involving or eligib* or decision or barrier* or facilitat* or ethic* or consent or acceptability or vulnerab* or engag* or regulat* or ethic*) N5 (pregnan* OR lactat* OR "breast feed*" OR breastfeed*) N5 (trials OR research) |
| 2 | ( pregnan* OR lactat* OR "breast feed*" OR breastfeed* ) AND ( "human research subjects" OR "patient selection" OR "patient participation" OR "medical experimentation on humans") |
| 3 | ( pregnan* OR lactat* OR "breast feed*" OR breastfeed* ) AND SU ( "clinical trials" OR "randomized controlled trials" OR "medical research") |
| 4 | (regulatory or regulations or decision or barrier* or facilitat* or ethic* or moral* or acceptability or vulnerab* or willing* or qualitative or altruis* or opportunit* or underrepresent* or unfair or fair or fairness or "scientifically complex" or stakeholder* or concerns or "focus group*" or incentive*) |
| 5 | TI ( challenge* or eligib* or consent or recommend* ) OR SU ( challenge* or eligib* or consent or recommend* ) OR KW ( challenge* or eligib* or consent or recommend* ) |
| 6 | represent* N5 (pregnan* or breastfeed* or breast feed* or lactating or lactation or women or minorit* or gender*) |
| 7 | perception* or perspective* or opinion* or views or viewpoint* or reasons or reason or experience or experiences |
| 8 | attitude* |
| 9 | policy or policies or initiative* or "pharmaceutical compan*" or "pharmaceutical industr*" or "drug compan*" or "drug industr*" or "research agenda" or rule or rules or law or laws or legal or legislation or governance or guidance or "research design" or "study design" or "research subjects" |
| 10 | TI ( guideline* or implementation or safety ) OR SU ( guideline* or implementation or safety ) OR KW ( guideline* or implementation or safety ) |
| 11 | "presumptive exclusion" or "unfair exclusion" |
| 12 | (participat* or inclusion or recruit or recruitment or enrol or enrolment or enrollment or involvement or involving or engage or engagement) N3 (pregnan* or lactating or lactation or breastfeed* or "breast feed*") |
| 13 | motivation or incentive* or participation or "decision making" or eligibility or acceptability or stakeholder* or ethic* or bioethic* |
| 14 | S4 OR S5 OR S6 OR S7 OR S8 OR S9 OR S10 OR S11 OR S12 OR S13 |
| 15 | S3 AND S14 |
| 16 | S1 OR S2 OR S15 |

Web of Science

| # | Searches |
| --- | --- |
| 1 | TI=(inclu* or exclu* or participat* or recruit* or enrol* or "involvement" or "involving" or eligib* or "decision" or barrier* or facilitat* or ethic* or "consent" or "acceptability" or vulnerab* or engag* or regulat* or ethic*) OR AK=(inclu* or exclu* or participat* or recruit* or enrol* or "involvement" or "involving" or eligib* or "decision" or barrier* or facilitat* or ethic* or "consent" or "acceptability" or vulnerab* or engag* or regulat* or ethic*) |
| 2 | TI=(pregnan* or "lactating" or "lactation" or breastfeed* or "breast feed*" or "women" or "gender") OR AK=(pregnan* or "lactating" or "lactation" or breastfeed* or "breast feed*" or "women" or "gender") |
| 3 | TI=("trials" or "trial" or "research" or "clinical studies") OR AK=("trials" or "trial" or "research" or "clinical studies") |
| 4 | #1 AND #2 AND #3 |
| 5 | TI=("pregnant" or "pregnancy" or "lactating" or "lactation" or breastfeed* or "breast feed*") OR AK=("pregnant" or "pregnancy" or "lactating" or "lactation" or breastfeed* or "breast feed*") |
| 6 | TI=(("drug" or "drugs" or pharmaceutical* or vaccine* or "medication" or "medications") NEAR/1 ("development" or "approval" or "regulation" or "evaluation")) OR AK=(("drug" or "drugs" or pharmaceutical* or vaccine* or "medication" or "medications") NEAR/1 ("development" or "approval" or "regulation" or "evaluation")) |
| 7 | TI=("trials" OR "biomedical research" OR "drug research") OR AK=("trials" OR "biomedical research" OR "drug research") |
| 8 | #5 AND (#6 or #7) |
| 9 | AB=((inclu* or exclu* or participat* or recruit* or enrol* or "involvement" or "involving" or eligib* or "decision" or barrier* or facilitat* or ethic* or "consent" or "acceptability" or vulnerab* or engag* or regulat* or ethic*) NEAR/5 (pregnan* or "lactating" or "lactation" or breastfeed* or "breast feed*") NEAR/5 ("trials" or "research" or "clinical studies")) |
| 10 | #4 OR #8 OR #9 |
| 11 | TS=(regulat* or challenge* or eligib* or "decision" or barrier* or facilitat* or ethic* or moral* or "consent" or "acceptability" or vulnerab* or willing* or determinant* or benefit* or "factor" or "factors" or "qualitative" or altruis* or opportunit* or represent* or underrepresent* or "risk" or "risks" or "unfair" or "fair" or "fairness" or "scientifically complex" or stakeholder* or "concerns" or "focus group*" or interview* or recommend* or incentive*) |
| 12 | TS=(perception* or perspective* or opinion* or "view" or" views" or viewpoint* or "reasons" or "reason" or attitude* or experience*) |
| 13 | TS=("policy" or "policies" or guideline* or model* or initiative* or "implementation" or "pharmaceutical compan*" or "pharmaceutical industr*" or "drug compan*" or "drug industr*" or "research agenda" or classif* or reclassif* or "rule" or "rules" or "law" or "laws" or "legal" or "governance" or "guidance" or "safety" or "assessment" or "research design" or "study design" or "research subjects") |
| 14 | TS=("presumptive exclusion" or "unfair exclusion") |
| 15 | TS=((participat* or inclu* or recruit* or enrol* or involv* or engag*) NEAR/4 (pregnan* or "lactating" or "lactation" or breastfeed* or "breast feed*")) |
| 16 | TS=("knowledge" or "attitude*" OR "motivation" OR "selection" OR "trust" or committee*) |
| 17 | #11 OR #12 OR #13 OR #14 OR #15 OR #16 |
| 18 | #10 AND #17(Exclude – Document Types: Meeting Abstract) |

Scopus

Scopus search strategy was based on the Web of Science search
