# Supplementary material for: Factors influencing the participation of pregnant and lactating women in clinical trials: A mixed-methods systematic review
Source: PLoS Med. 2024 May 30;21(5):e1004405. doi: 10.1371/journal.pmed.1004405 (PMC11139290; doi:10.1371/journal.pmed.1004405)
Supplement: S4 Appendix — (DOCX) [file pmed.1004405.s004.docx]

S4. Appendix: GRADE-CERQual Evidence Profile

| **Findings** | **Summary of qualitative review findings** | **Contributing qualitative studies** | **Methodological limitations** | **Coherence** | **Relevance** | **Adequacy** | **Overall CERQual assessment** | **Explanation of overall assessment** |
| --- | --- | --- | --- | --- | --- | --- | --- | --- |
|  | **Interplay between perceived risks and benefits of participation in women’s decision-making** | | | | | | | |
| 1 | **Women have a limited appetite for risk during pregnancy or lactation**  Perception of risks influenced pregnant and lactating women's willingness to participate in trials, which varied based on their individual levels of risk tolerance, previous trial experiences, observations of others' experiences, stage of pregnancy or lactation, existing health conditions, and a sense of responsibility for their health and that of the fetus/infant. Women were more likely to decline participation if the experimental intervention was previously untested and were more confident to participate when convinced of no harm. | [39, 40, 47, 48, 57, 58, 60, 63-65, 69, 72, 74, 83, 84, 87, 89, 91, 92, 96] | Minor concerns:  14 papers with no or very minor issues, 3 papers with minor issues (recruitment/data collection, analysis, ethics, reflexivity), 2 papers with some issues (recruitment/data collection, analysis, coherence on designs, reflexivity), and 1 paper with substantial issues (recruitment/data collection, analysis, link from data to findings, coherence on designs, ethics, reflexivity) | No or very minor concerns | No or very minor concerns: All 20 papers directly relevant to the review aim. Contributing papers represented 4 regions, WPRO (New Zealand, Australia), AFRO (Ghana, Malawi, Liberia), EURO (France, Germany, Italy, Spain, United Kingdom, Ireland, Netherlands) and PAHO (United States, Canada), where 16 are high income countries, 1 is lower middle income country, 2 are low income countries. Perspectives came from pregnant, postpartum and lactating women, health workers, ethics committee members, research staff, study investigators and regulators. Focal population is pregnant and lactating women | No or very minor concerns: 20 papers contributed to review finding with relatively thick data | High confidence | No or very minor concerns on relevance, no or very minor concerns on coherence, no or very minor concerns on adequacy, minor concerns on methodological limitations (recruitment/data collection, analysis, link from data to findings, coherence on designs, ethics, reflexivity) |
| 2 | **Making trade-offs between risk and severity of the condition and risk-benefit ratio of intervention**  Before participating, women weighed the risk of their medical condition and its impact, especially on the baby, against the risks of an intervention and its potential benefits. Women were less likely to participate if they felt healthy or perceived themselves at low risk of experiencing or being negatively affected by the condition, believed they had nothing to gain from participating, or felt concerned that the intervention risks were too high. | [39, 48, 57-60, 63, 64, 69, 72, 74, 87, 91, 96] | No or very minor concerns: 10 papers with no or very minor issues, 2 papers with minor issues (recruitment/data collection, analysis, ethics, reflexivity), and 2 papers with some issues (recruitment/data collection, analysis, coherence on designs, reflexivity) | No or very minor concerns | Moderate concerns: 13 out 14 papers directly relevant to review aim. Contributing papers represented 3 regions - EURO (France, Germany, Italy, Spain and the United Kingdom, Netherlands), PAHO (United States, Canada), 9 countries where all are high income countries.  Perspectives came from pregnant and lactating women, health workers and study investigators. Focal population are pregnant and lactating women | Minor concerns: 14 papers contributed to review finding with relatively thick data | Moderate confidence | No or very minor concerns on methodological limitations, no or very minor concerns on coherence, minor concerns on adequacy (14 papers relatively thick data), moderate concerns on relevance (all are high income countries) |
| 3 | **Benefits to health arising from participation**  A key motivating factor for pregnant and lactating women to participate in trials was the expectation of personal health benefits, such as improved knowledge about how the condition affected them, protecting their fetus or infant from harm, and reducing mother-to-child disease transmission. When women saw the potential for these benefits, deciding not to participate was viewed as potentially putting the baby's life at risk. | [40, 47, 55, 60, 61, 63, 64, 70, 73, 83, 84, 87, 90-92, 96] | Minor concerns: 8 papers with no or very minor issues, 4 papers with minor issues (recruitment/data collection, coherence on designs, ethics, reflexivity), 2 papers with some issues (recruitment/data collection, analysis, coherence on designs, reflexivity) and 2 papers with substantial issues (recruitment/data collection, analysis, link from data to findings, coherence on designs, ethics, reflexivity) | No or very minor concerns | No or very minor concerns: 16 out of 16 papers directly relevant to the review aim. Contributing papers represented 4 regions – EURO (United Kingdom, Spain, Netherlands, Belgium, Ireland), PAHO (Brazil, United States, Canada), AFRO (Ghana, Malawi) and WPRO (New Zealand, Australia), 12 countries where 9 are high income countries, 1 is upper middle-income country, 1 is lower middle-income country, and 1 is low-income country country. Perspectives came from pregnant, postpartum and lactating women, women who had breastfed within the past 5 years, partners, health workers, research staff, study investigators and ethics committee members. Focal populations are pregnant and lactating women. | Minor concerns: 16 papers contributed to review finding with relatively thick data | High confidence | No or very minor concerns on relevance, no or very minor concerns on coherence, minor concerns on methodological limitations (recruitment/data collection, analysis, link from data to findings, coherence on designs, ethics, reflexivity), minor concerns on adequacy (16 papers with relatively thick data) |
| 4 | **Experiences and expectations of high-quality care motivate participation**  Pregnant and lactating women were motivated to participate as a token of appreciation to health workers who provided good quality care. Additionally, women were more likely to participate when they perceived that it would result in higher quality clinical care or access to vaccines or therapeutic products that had previously been denied or were otherwise not accessible outside the context of a trial. | [39, 48, 49, 60, 63, 70, 72, 83, 84, 86, 87, 92, 96] | Minor concerns: 7 papers with no or very minor issues, 4 papers with minor issues (recruitment/data collection, analysis, link from data to findings, coherence on designs, ethics, reflexivity) and 2 papers with some issues (recruitment/data collection, analysis, coherence on designs, ethics, reflexivity) | No or very minor concerns | No or very minor concerns: 12 out of 13 papers directly relevant to the review aim. Contributing papers represented 4 regions - EURO (United Kingdom, Republic of Ireland), PAHO (Brazil, United States), AFRO (Malawi) and WPRO (Australia), and 6 countries where 4 are high income countries, 1 is upper middle-income country and 1 is a low-income country. Perspective came from pregnant, postpartum and lactating women, women who had breastfed within the past 5 years and research staff. Focal populations are pregnant and lactating women. | Minor concerns: 13 papers contributed to this review finding, with relatively thin data | High confidence | No or very minor concerns on relevance, no or very minor concerns on coherence, minor concerns on adequacy (13 papers with relatively thin data), minor concerns on methodological limitations (recruitment/data collection, analysis, link from data to findings, coherence on designs, ethics, reflexivity) |
| 5 | **Knowledge of the rationale for study design features**  The rationale behind certain trial design features such as randomisation, blinding or inclusion of a placebo arm could be a source of confusion, concern, or reassurance for potential participants, impacting their decisions to participate. These features could be viewed as preferential treatment of one group over another, adding burden with little opportunity for personal benefit, a mechanism to reduce bias or conversely for researchers to avoid accountability for an adverse outcome. | [39, 40, 45, 59, 62, 63, 69, 72, 74, 87, 91, 92] | Minor concerns: 7 papers with no or very minor issues, 4 papers with minor issues (recruitment/data collection, analysis, ethics, reflexivity), and 1 paper with some issues (recruitment/data collection, analysis, coherence on designs, reflexivity) | No or very minor concerns | Moderate concerns:  12 out of 12 papers directly relevant to review aim. Contributing papers represented 4 regions - EURO (United Kingdom, Netherlands, Republic of Ireland), PAHO (United States, Canada), WPRO (New Zealand, Australia) and AFRO (Malawi), and 8 countries - 7 countries are high income, and 1 country is low-income.  Perspectives came from pregnant and postpartum women, health workers and study investigators. Focal population is pregnant and lactating women. | Minor concerns: 12 papers contributed to this review finding, with relatively thick data | Moderate confidence | No or very minor concerns on coherence, minor concerns on adequacy (12 papers contributed with relatively thick data), minor concerns on methodological limitations (recruitment/data collection, analysis, ethics and reflexivity), moderate concerns on relevance (no papers conducted in middle-income countries and only one paper in a low-income country) |
| 6 | **Acceptability of the intervention is key to pregnant and lactating women's willingness to participate in a trial, and for research staff to recruit for a trial**  Interventions that were most acceptable to women and research staff were those that simplified intervention delivery, were less onerous or painful than usual care, had negligible risk, were non-invasive, placed limited demands on time, did not involve invasive procedures, and where prior knowledge about the condition intersected with positive attitudes towards the therapeutic product. | [40, 45, 48, 53, 54, 61, 64, 65, 72, 73, 81, 83, 86, 87, 90-92, 96] | No or very minor concerns: 13 papers with no or very minor issues, 3 papers with minor issues (recruitment/data collection, link from data to findings, ethics, reflexivity) and 2 papers with some issues (recruitment/data collection, analysis, coherence on designs, reflexivity). | No or very minor concerns | Minor concerns: 17 out of 18 papers directly relevant to the review aim. Contributing papers represented 4 regions - EURO (Republic of Ireland, United Kingdom, Spain, Netherlands), PAHO (Canada), WPRO (New Zealand, Australia) and AFRO (Ghana, Malawi, Liberia), and 10 countries - 7 countries are high income, and 1 country is lower-middle income, and 2 countries are low-income.  Perspectives came from pregnant and postpartum women, lactating women, health workers, research staff, study investigators and ethics committee members. Focal population is pregnant and lactating women. | Minor concerns: 18 papers contributed to this review finding, with relatively thick data | High confidence | No or very minor concerns on methodological limitations, no or very minor concerns on coherence, minor concerns on adequacy (18 papers with relatively thick data), minor concerns on relevance (no papers conducted in upper-middle-income countries) |
| 7 | **Fears around data sharing and use**  Some women feared that trial participation, including provision of blood samples, could expose them to stigmatisation and judgement due to unwanted diagnoses and disclosure of disease status, data sharing regarding sensitive behaviours, and the threat of their data being used in ways that would compromise confidentiality and safety. | [65, 85, 86] | No or very minor concerns: 2 papers with no or very minor issues and 1 paper with minor issues (link from data to findings, reflexivity) | No or very minor concerns | Moderate concerns: 1 out of 3 papers directly relevant to the review aim. Contributing papers represented 3 regions - EURO (United Kingdom), PAHO (United States) and AFRO (Liberia, Malawi), and 4 countries - 2 countries are high income and 2 countries are low-income.  Perspectives came from pregnant women, postpartum women, health workers and research staff. Focal population is pregnant women | Serious concerns: 3 papers contributed to this review finding, with moderately thin data. | Low confidence | No or very minor concerns on methodological limitations, no or very minor concerns on coherence, moderate concerns on relevance (2 papers indirectly relevant to review aim, and no representation from middle-income countries), serious concerns on adequacy (3 papers with moderately thin data) |
| 8 | **Altruistic motivations**  Pregnant women expressed willingness to participate in trials for the purpose of contributing to societal benefits of research, including the potential to improve health and healthcare for pregnant women in the future. Altruistic motivations could act as a stand-alone stimulus, secondary to or alongside beliefs around personal benefit, or conditional on no additional risk for participation. | [39, 40, 47, 48, 55-61, 63, 64, 70, 72-74, 83, 86, 87, 89, 91, 92] | Moderate concerns: 13 papers with no or very minor issues, 6 papers with minor issues (recruitment/data collection, analysis, link from data to findings, coherence on designs, ethics, reflexivity), 2 papers with some issues (recruitment/data collection, analysis, coherence on designs, reflexivity), and 2 papers with substantial issues (recruitment/data collection, analysis, link from data to findings, coherence on designs, ethics, reflexivity) | No or very minor concerns | Minor concerns: 22 out of 23 papers directly relevant to review aim. Contributing studies represented 4 regions - EURO (United Kingdom, Netherlands, France, Germany, Italy, Spain, Belgium, Republic of Ireland), PAHO (United States, Canada, Brazil), WPRO (New Zealand, Australia) and AFRO (Ghana), and 14 countries - 12 countries are high income, 1 country is upper middle income, and 1 country is lower-middle income.  Perspectives came from pregnant and postpartum women, partners, health workers, research staff, study investigators, ethics committee members and regulators. Focal population is pregnant women. | Minor concerns: 23 papers contributed to this review finding, with relatively thin data | Moderate confidence | No or very minor concerns on coherence, minor concerns on relevance (no studies conducted in low-income countries), minor concerns on adequacy (23 papers with relatively thin data), moderate concerns on methodological limitations (recruitment/data collection, analysis, link from data to findings, coherence on designs, ethics and reflexivity) |
| 9 | **Financial incentives**  Pregnant and lactating women had mixed attitudes to financial incentives for research participation. Some viewed financial incentives as acceptable, with higher remuneration as an appropriate strategy to encourage participation, whereas others viewed financial incentives as potentially coercive, especially in the context of poverty. Some women felt that financial reimbursements did not play a substantial role in women’s decision-making. | [39, 55, 65, 83, 96] | Minor concerns: 3 papers with no or very minor issues, 1 paper with minor issues (recruitment/data collection, analysis, ethics, reflexivity) and 1 paper with substantial issues (analysis, link from data to findings, coherence on designs, ethics, reflexivity). | No or very minor concerns | Moderate concerns: 5 out of 5 papers directly relevant to the review aim. Contributing papers represented 2 regions - EURO (United Kingdom, Belgium) and AFRO (Liberia), and 3 countries - 2 countries are high income, and 1 country is low-income.  Perspectives came from pregnant and postpartum women, lactating women and health workers. Focal population is pregnant and lactating women | Serious concerns: 5 papers contributed to this review finding, with mostly thin data | Low confidence | No or very minor concerns on coherence, minor concerns on methodological limitations (recruitment/data collection, analysis, link from data to findings, coherence on designs, ethics, reflexivity), moderate concerns on relevance (no papers conducted in middle-income countries, and only 2 WHO regions and 3 countries represented), serious concerns on adequacy (5 papers with mostly thin data) |
|  | **Engagement between women and the medical and research ecosystems** | | | | | | | |
| 10 | **Roles of trust and power in the medical and research ecosystem**  Pregnant and lactating women's willingness to participate in trials was driven by trust, confidence, and faith in medicine and research, and women relied on the opinions of the health workers that they consulted with regarding the efficacy and safety of the intervention. Simultaneously, power imbalances between women and health workers, coupled with women's therapeutic misconceptions, could lead to coercion in participation. This ethical dilemma was recognized by study investigators, ethics committee members, and women, especially in the context of the dual roles of clinician-researchers; however, power and credibility when combined with good rapport and clear communication generated trust to participate or comfort to decline. While rare, some women had larger concerns about the vested interests of pharmaceutical companies. | [39, 40, 42-45, 47-49, 56-61, 65, 69, 70, 72-74, 81, 82, 86, 87, 89, 91, 92] | Minor concerns: 18 papers with no or very minor issues, 5 papers with minor issues (recruitment/data collection, analysis, link from data to findings, coherence on designs, ethics, reflexivity), 3 papers with some issues (recruitment/data collection, analysis, coherence on designs, ethics, reflexivity), and 3 papers with substantial issues (recruitment/data collection, analysis, link from data to findings, coherence on designs, ethics, reflexivity) | No or very minor concerns | No or very minor concerns: 26 out of 28 papers directly relevant to review aim. Contributing papers represented 4 regions - EURO (United Kingdom, Netherlands, France, Germany, Italy, Spain, Republic of Ireland ), PAHO (United States, Canada, Brazil), WPRO (New Zealand, Australia) and AFRO (Ghana, Malawi, Benin, The Gambia and Burkina Faso, Liberia), and 18 countries - 11 countries are high income, 1 country is upper middle income, 2 countries are lower middle income, and 4 countries are low-income.  Perspectives came from pregnant women, postpartum women, partners, community leaders, traditional healers, traditional birth attendants, health workers, study investigators, research staff, ethics committee members and regulators. Focal population is pregnant and lactating women | Minor concerns: 28 papers contributed to this review finding, with relatively thick data | High confidence | No or very minor concerns on relevance, no or very minor concerns on coherence,  minor concerns on methodological limitations (recruitment/data collection, analysis, link from data to findings, coherence on designs, ethics, reflexivity), minor concerns on adequacy (28 papers contributed to review findings with relatively thick data) |
| 11 | **The role of therapeutic hope and optimism**  Therapeutic hope and optimism played a critical role for health workers and research staff to administer trials, and for pregnant and lactating women to participate in trials. Prior knowledge about and experience with using the intervention, observation of potential beneficial effects, and trust in health workers shaped feelings of therapeutic hope and optimism. However, for some women, a lack of understanding of the differences between research and clinical care when combined with therapeutic hope led to therapeutic misconceptions and unmet expectations about the personal benefits arising from trial participation. | [42, 45, 47, 53, 65, 70, 74, 81, 82, 87] | Moderate concerns: 6 papers with no or very minor issues, 1 paper with minor issues (recruitment/data collection, coherence on designs, ethics, reflexivity), , 1 paper with some issues (recruitment/data collection, analysis, coherence on designs, reflexivity) and 2 papers with substantial issues (recruitment/data collection, analysis, link from data to findings, coherence on designs, ethics, reflexivity) | No or very minor concerns | No or very minor concerns: All included papers directly relevant to review aim. Contributing papers represented 34 regions, EURO (United Kingdom, Netherlands), PAHO (United States, Brazil), AFRO (Ghana, Malawi, Liberia), and WPRO (Australia), 8 countries where 4 are high income countries, 1 is upper middle income country, 1 lower middle income country and 2 low income country. Perspectives came from health workers, research staff, study investigators, pregnant and lactating women, partners, family and community members. Focal populations are pregnant and lactating women | Moderate concerns: 10 papers contributed to review finding with moderately thin data | Moderate confidence | No or very minor concerns on coherence, no or very minor concerns on relevance, moderate concerns on adequacy (10 papers contributed with relatively thin data), moderate concerns on methodological limitations (recruitment/data collection, coherence on designs, ethics, reflexivity, analysis, link from data to findings) |
|  | **Gender norms and decision-making autonomy** | | | | | | | |
| 12 | **Expectations of women’s roles as mothers and caregivers**  Pregnant and lactating women's decisions to participate in clinical trials were often influenced by their strong sense of responsibility towards the health and care of their fetus or infant, themselves, and their families. This sense of responsibility was endorsed and reinforced by familial and societal expectations of what it means to be a good mother. | [60, 61, 64, 91, 96] | No or very minor concerns: 4 papers with no or very minor issues and 1 paper with some issues (recruitment/data collection, analysis, coherence on designs, reflexivity) | No or very minor concerns | Moderate concerns: 5 out of 5 papers directly relevant to the review aim. Contributing papers represented 2 regions - EURO (United Kingdom, Spain) and PAHO (United States, Canada), and 4 countries all of which are high income.  Perspectives came from pregnant women, postpartum women, lactating women, women who had breastfed within the past 5 years, health workers and research staff, and study investigators. Focal population is pregnant and lactating women | Serious concerns: 5 papers contributed to this review finding, with relatively thin data | Low confidence | No or very minor concerns on methodological limitations, no or very minor concerns on coherence, serious concerns on adequacy (5 papers with relatively thin data), moderate concerns on relevance (No representation from low-and middle-income countries) |
| 13 | **Role of bodily autonomy in decision-making**  Some women, health workers, ethics committee members and regulators perceived that pregnant women might not be able to make decisions by themselves about trial participation due to fetal involvement, inability to make rational choices during pregnancy, hormones, the stressful context of hospitalisation and financial inducements. However, research staff and some women believed in the right to bodily autonomy to make decisions by themselves despite having discussions with partners, family members, support persons or health workers. Women viewed other people making decisions regarding their participation as a violation of this right, though some women declined participation due to pressure from family members. | [39, 40, 43, 47, 54, 56, 72, 74, 81, 82, 85, 87, 90, 92] | Minor concerns: 10 papers with no or very minor issues, 2 papers with minor issues (recruitment/data collection, analysis, coherence on designs, ethics, reflexivity), 1 paper with some issues (recruitment/data collection, analysis, coherence on designs, reflexivity) and 1 paper with substantial issues (recruitment/data collection, analysis, link from data to findings, coherence on designs, ethics, reflexivity). | No or very minor concerns | Minor concerns: 13 out of 14 papers directly relevant to review aim. Contributing papers represented 4 regions: EURO (United Kingdom, Ireland, Netherlands), PAHO (United States), AFRO (Ghana, Malawi), WPRO (New Zealand, Australia), 8 countries where 6 are high income countries, 1 is lower middle income country and 1 is low income country. Perspectives came from pregnant and postpartum women, partners, health workers, research staff, study investigators, regulators and ethics committee members. Focal population is pregnant and lactating women | Minor concerns: 14 papers contributed to review finding with moderately thick data | Moderate confidence | No or very minor concerns on coherence, minor concerns on relevance (13 out 14 papers directly relevant to review aim), minor concerns on methodological limitations (recruitment/data collection, analysis, link from data to findings, coherence on designs, ethics, reflexivity), minor concerns on adequacy (14 papers with moderately thick data) |
| 14 | **Relationship dynamics, gender roles and norms are key to women’s attitudes to partner involvement and paternal consent**  Pregnant women often discussed the benefits and risks of trial participation with their partners – especially in the context of fetal involvement – and their final decision may or may not have been influenced by their partners**'** own attitudes. In some settings, pregnant women**'**s trial participation was contingent on partners**'** buy-in, and the formality justified in the context of gender norms and roles. These could be the partner being the household head, to allay men’s suspicions about women’s whereabouts and interactions, and to minimise any misunderstanding related to positive tests or disease status that might cast doubt on women’s fidelity to their husbands. | [39, 40, 42, 43, 47, 60, 64, 65, 69, 72, 74, 81, 83, 85, 87, 90, 91] | Minor concerns: 11 papers with no or very minor issues, 2 papers with minor issues (recruitment/data collection, analysis, ethics, reflexivity), and 2 papers with some issues (recruitment/data collection, analysis, coherence on designs, reflexivity), and 2 papers with substantial issues (recruitment/data collection, analysis, link from data to findings, coherence on designs, ethics, reflexivity) | No or very minor concerns | No or very minor concerns: 16 out of 17 papers directly relevant to review aim. Contributing papers represented 4 regions: EURO (United Kingdom, Netherlands, Spain), AFRO (Ghana, Malawi, Liberia), PAHO (United States, Canada), WPRO (New Zealand, Australia), 10 countries where 7 are high income countries, 1 is lower middle income country and 2 are low income countries. Perspectives came from pregnant, lactating and postpartum women, health workers, research staff, study investigators, regulators, ethics committee members and community leaders. Focal population is pregnant women | Moderate concerns: 17 papers contributed to review finding with relatively thin data | Moderate confidence | No or very minor concerns on coherence, no or very minor concerns on relevance, minor concerns on methodological limitations (recruitment/data collection, analysis, link from data to findings, coherence on designs, ethics, reflexivity), moderate concerns on adequacy (17 papers contributed with relatively thin data) |
|  | **Factors affecting clinical trial recruitment** | | | | | | | |
| 15 | **Developing trusting and reciprocal relationships with the community as part of the research process**  Designing and embedding research within communities required engaging with community norms, beliefs and practices. Some community members expressed how they viewed research negatively in the context of historical and ongoing oppressions that people experience due to colonisation, corruption, extractive practices, and civil and political conflict. Central to the acceptability and cultural safety of the research were investments in developing trusting relationships with community representatives and leaders. | [44, 45, 60, 65, 66, 74, 78, 83, 90, 92] | Minor concerns: 7 papers with no or very minor issues,1 paper with minor issues (analysis, reflexivity), and 2 paers with some issues (recruitment/data collection, analysis,coherence on designs, ethics, reflexivity) | No or very minor concerns | No or very minor concerns: 9 out of 10 papers directly relevant to review aim. Contributing papers represented 3 regions: EURO (United Kingdom, Netherlands), AFRO (Benin, The Gambia, Burkina Faso, Liberia, Ghana, Mali, Guinea, Nigeria, Sierra Leone, Gabon, Congo-Brazzaville, Malawi), PAHO (Unites States), 15 countries where 3 are high income countries, 1 is upper middle income country, 5 are lower middle income countries and 6 are low income countries. Perspectives came from pregnant and postpartum women, health workers, research staff, study investigators and community leaders. Focal population is pregnant and lactating women | Moderate concerns: 10 papers contributed to review finding with relatively thin data | Moderate confidence | No or very minor concerns on coherence, no or very minor concerns on relevance, minor concerns on methodological limitations (recruitment/data collection, analysis,coherence on designs, ethics, reflexivity), moderate concerns on adequacy (10 papers contributed with relatively thin data) |
| 16 | **Increasing visibility and awareness of the trial**  Increasing visibility and awareness of the trial to potential participants, health workers and community representatives influenced trial recruitment. Recommended strategies included paper and electronic promotional materials, regular physical presence of research staff in the areas where recruitment was taking place, and reminders to health workers about recruitment pathways and trial protocols through trainings. | [54, 62, 65, 74, 87] | Minor concerns: 3 papers with no or very minor issues, 1 paper with minor issues (recruitment/data collection, reflexivity) and 1 paper with some issues (recruitment/data collection, analysis, coherence on designs, reflexivity) | No or very minor concerns | Moderate concerns: 5 papers directly relevant to review aim. Contributing papers represented 3 regions: EURO (Ireland, United Kingdom, Netherlands), AFRO (Liberia),and WPRO (Australia), 5 countries where 4 are high income countries, and 1 is low income country. Perspectives came from pregnant women, research staff, study investigators health workers, and community leaders.  Focal populations are pregnant and lactating women. | Serious concerns: 5 papers contributed to review findings with relatively thick data | Low confidence | No or very minor concerns on coherence, minor concerns on methodological limitations (recruitment/data collection, analysis, coherence on designs, reflexivity), moderate concerns on relevance (contributing papers represented 3 regions where 4 are high income countries and 1 low income country), serious concerns on adequacy (5 papers contributed with relatively thick data) |
| 17 | **Inadequate resources**  Inadequate physical infrastructure, time, finances, and insufficient quantity and quality of human resources were barriers for research staff to recruit women for clinical trials. For health workers specifically, heavy workloads made it challenging to incorporate trial recruitment into clinical workflows, and the added burden and sometimes insufficient compensation, contributed to poor morale. | [44, 54, 55, 62, 87, 89] | Moderate concerns: 2 papers with no or very minor issues, 1 study with minor issues (recruitment/data collection, reflexivity), and 2 papers with some issues (recruitment. data collection, analysis, coherence on designs, ethics, reflexivity), 1 paper with substantial issues (analysis, link from data to findings, coherence on designs, ethics, reflexivity) | No or very minor concerns | No or very minor concerns: 5 out of 6 papers directly relevant to review aim. Contributing papers represented 3 regions: EURO (Ireland, United Kingdom, Belgium, Netherlands), AFRO (Benin, The Gambia, Burkina Faso), and WPRO (Australia), 8 countries where 5 are high income countries, 1 is lower middle income country and 2 are low income countries. Perspectives came from health workers, research staff and study investigators.  Focal populations are pregnant and lactating women. | Serious concerns: 6 papers contributed to review finding with relatively thin data | Low confidence | No or very minor concerns on coherence, no or very minor concerns on relevance, moderate concerns on methodological limitations (recruitment/data collection, analysis, link from data to findings, coherence on designs, ethics, reflexivity), serious concerns on adequacy (6 papers contributed with relatively thin data), |
| 18 | **Engaging health workers in trials**  Research staff perceived the importance of building reciprocal and collaborative relationships with health workers because some acted as gatekeepers. Some health workers, however, were reluctant to engage women in clinical trials due to a lack of knowledge about trial design and the research value, varying levels of acceptability of risk, perceived obligation to protect women, and a lack of trust in the research team. Health workers supported inclusion when trial protocols included close monitoring of risks, and when there was clinical equipoise alongside therapeutic hope in the trial intervention. These factors were informed by their clinical knowledge, previous clinical experiences using the intervention, and observed outcomes in the current trial. | [47, 53-55, 60, 62, 64, 65, 87, 89-91] | Minor concerns: 7 papers with no or very minor issues, 1 paper with minor issues (recruitment/data collection, reflexivity), 2 papers with some issues (recruitment/data collection, analysis, coherence on designs, reflexivity), and 2 papers with substantial issues (recruitment/data collection, analysis, link from data to findings, coherence on designs, ethics reflexivity) | No or very minor concerns | Minor concerns: 12 papers directly relevant to review aim. Contributing papers represented 4regions: EURO (United Kingdom, Netherlands, Ireland, Spain, Belgium), AFRO (Liberia, Ghana), PAHO (Unites States, Canada), and WPRO (Australia), 10 countries where 8 are high income countries, 1 is lower middle income country and 1 is low income country. Perspectives came from health workers, research staff and study investigators.  Focal populations are pregnant and lactating women | Minor concerns: 12 papers contributed to review finding with thick data | High confidence | No or very minor concerns on coherence, minor concerns on adequacy (12 papers contributed with thick data), minor concerns on relevance (contributing papers represented 10 countries with 8 high income and 1 lower middle income and 1 low income country), minor concerns on methodological limitations (recruitment/data collection, analysis, link from data to findings, coherence on designs, ethics reflexivity) |
| 19 | **Research staff's emotional orientations towards clinical trials**  Having a sense of trial ownership, supportive teamwork, a shared sense of team achievement and motivation to achieve recruitment targets could support successful trial recruitment. However, feeling pressured by the recruitment process, seeing it as a procedural activity and needing to implement complex study designs impacted research staffs**'** ability to recruit women, leading to frustration and lower enthusiasm. | [53, 54, 62] | No or very minor concerns: 2 papers with no or very minor issues, 1 paper with minor issues (recruitment/data collection, reflexivity) | No or very minor concerns | Serious concerns: 3 papers directly relevant to review aim. Contributing papers represented 1 region: EURO (United Kingdom, Ireland), 2 countries where all are high income countries. Perspectives came from health workers, and research staff. Focal populations are pregnant and lactating women | Moderate concerns: 3 papers contributed to review finding with thick data | Low confidence | No or very minor concerns on coherence, no or very minor concerns on methodological limitations, moderate concerns on adequacy (3 papers with thick data), serious concerns on relevance (papers represented 1 region where all countries are high income countries) |
| 20 | **Women-centred approach encourages participation**  Women valued an individualised, humanised, and transparent approach to communication, and adequate time during trial recruitment to discuss details and concerns related to the trial. These helped ensure they had sufficient capacity and opportunity to make informed decisions. Similarly, research staff found that approaching potential participants at the ‘right time’ and in an appropriate manner by considering their physical and mental state, providing adequate information and engaging in discussions increased recruitment success t. | [39, 40, 54, 56, 62, 66, 69, 70, 72, 74, 86, 87, 92] | Minor concerns: 6 papers with no or very minor issues, 6 papers with minor issues (recruitment/data collection, analysis, link from data to findings, coherence on designs, ethics, reflexivity) and 1 paper with some issues (recruitment/data collection, analysis, coherence on designs, reflexivity) | No or very minor concerns | Moderate concerns: 12 out of 13 papers directly relevant to the review aim. Contributing papers represented 3 regions - EURO (United Kingdom, Republic of Ireland, Netherlands), PAHO (Brazil, United States) and WPRO (New Zealand, Australia), and 7 countries - 6 countries are high income, and 1 country is upper middle-income. Perspectives came from pregnant and lactating women, health workers, study investigators, and research staff. Focal population is pregnant and lactating women. | Minor concerns: 13 papers contributed to this review finding, with mostly thick data | Moderate confidence | No or very minor concerns on coherence, minor concerns on methodological limitations (recruitment/data collection, analysis, link from data to findings, coherence on designs, ethics, reflexivity), minor concerns on adequacy (13 papers contributed with mostly thick data), moderate concerns on relevance (No representation from low-income countries) |
| 21 | **Recruitment for intrapartum research**  Pain, intensity, and duration of labour motivated pregnant women to participate in intrapartum clinical trials. However, women, their partners, and research staff recognised the challenges in ensure women make informed decisions during this sensitive time, as decisions had to be made quickly, and partners were reluctant to make decisions on women’s behalf, even during emergencies, due to fears of negative outcomes. To optimise women making informed decisions, research staff provided information clearly and succinctly during the intrapartum period and tried to offer adequate time for decision-making. Most women recommended having trial information provided in the antenatal period, and revisiting trial details, including having a de-briefing about one's own experience, prior to discharge. | [43, 49, 56, 59, 61, 62, 81, 82, 86, 91] | Minor concerns: 7 papers with no or very minor issues, 2 papers with minor issues (link from data to findings, recruitment/data collection, reflexivity), and 1 paper with some issues (recruitment/data collection, analysis, link from data to findings, coherence on designs, ethics, reflexivity) | No or very minor concerns | Moderate concerns: 9 out of 10 papers directly relevant to review aim. Contributing papers represented 2 regions, PAHO (United States, Canada) and EURO (United Kingdom), 3 countries where all are high income countries. Perspectives came from pregnant and lactating women, health workers, research staff and study investigators. Focal population is pregnant women | Minor concerns: 10 papers contributed to review finding with relatively thick data | Moderate confidence | No or very minor concerns on coherence, minor concerns on adequacy (10 papers contributed with relatively thick data), minor concerns on methodological limitations (recruitment/data collection, analysis, link from data to findings, coherence on designs, ethics, reflexivity), moderate concerns on relevance (9 out of 10 papers indirectly relevant, all papers are high income countries) |
|  | **Upstream factors affecting the research ecosystem** | | | | | | | |
| 22 | **Factors affecting motivation of study investigators**  The underlying factors that motivated many study investigators to conduct research with pregnant women were ethical responsibility, passion towards equity, and dedication to improving women’s health status and care, and filling scientific gaps. Additionally, lived experience of being pregnant, having mentors in this area in early careers, and previous research experiences with pregnant women contributed to study investigators’ motivations. However, concerns about risks of teratogenicity demotivated some investigators. | [42, 43, 66, 78, 89, 91] | Moderate concerns: 3 papers with no or very minor issues, 1 paper with minor issues (analysis, reflexivity), 1 paper with some issues (recruitment/data collection, link from data to findings, ethics, reflexivity), 1 paper with substantial issues (analysis, link from data to findings, coherence on designs, ethics, reflexivity) | No or very minor concerns | No or very minor concerns: 5 papers directly relevant to review aim. Contributing papers represented 3 regions: PAHO (United States, Canada), EURO (Netherlands), AFRO (Benin, Burkina Faso, Ghana, Mali, Guinea, Nigeria, Sierra Leone, Gabon, Congo-Brazzaville), 12 countries where 3 are high income countries, 1 is upper middle income country, 5 are lower middle income countries, and 3 are low income countries. Perspectives came from study investigators. Focal populations are pregnant women. | Minor concerns: 6 papers contributed to review finding with relatively thick data | Moderate confidence | No or very minor concerns on coherence, no or very minor concerns on relevance, minor concerns on adequacy (6 papers contributed with relatively thick data), moderate concerns on methodological limitations ( recruitment/data collection, analysis, link from data to findings, coherence on designs, ethics, reflexivity) |
| 23 | **Challenges in gaining ethical approvals for trials with pregnant women**  While some regulators, ethics committee members, and study investigators strongly support inclusion of pregnant women in clinical trials, most stakeholders start from a presumption of minimal risk to the fetus. This results in women**'**s exclusion, especially in the context of poor public stewardship, ambiguous guidelines, insufficient data on intervention safety, complexities and subjectivities in risk assessment, poor agreement on appropriate trial design, time consuming ethical processes, and concerns about reputation. | [42, 43, 66, 78, 82, 89-91] | Minor concerns: 5 papers with no or very minor issues, 1 paper with minor issues (analysis, reflexivity), 1 paper with some issues (recruitment/data collection, link from data to findings, ethics, reflexivity), 1 paper with substantial issues (analysis, link from data to findings, coherence on designs, ethics, reflexivity) | No or very minor concerns | No or very minor concerns: 8 papers directly relevant to review aim. Contributing papers represented 3 regions: PAHO (United States, Canada), EURO (United Kingdom, Netherlands), AFRO (Benin, Burkina Faso, Ghana, Mali, Guinea, Nigeria, Sierra Leone, Gabon, Congo-Brazzaville), 13 countries where 4 are high income countries, 1 is upper middle income country, 5 are lower middle income countries, and 3 are low income countries. Perspective came from study investigators, pregnant women, ethics committee members and regulators. Focal population is pregnant women | Minor concerns: 8 papers contributed to review finding with relatively thick data | Moderate confidence | No or very minor concerns on coherence, no or very minor concerns on relevance, minor concerns on adequacy (8 papers contributed with relatively thick data), minor concerns on methodological limitations (recruitment/data collection, analysis, link from data to findings, coherence on designs, ethics, reflexivity) |
| 24 | **Role of funders**  Limited interest of public and private funders and pharmaceutical companies to financially invest in trials due to the ethical complexities, potential for adverse events, liability, and possibility of political fallout was a barrier to conduct trials with pregnant and lactating women. When funding was available, funders' requests might facilitate the inclusion of pregnant women or create ethical challenges in conducting trials. | [54, 62, 66, 78] | Moderate concerns: 1 paper with no or very minor issues, 2 papers with minor issues (recruitment/data collection, analysis, reflexivity), 1 paper with some issues (recruitment/data collection, link from data to findings, ethics, reflexivity) | No or very minor concerns | No or very minor concerns: 4 papers directly relevant to review aim. Contributing papers represented 3 regions: PAHO (United States), EURO (United Kingdom, Ireland), AFRO (Benin, Burkina Faso, Ghana, Mali, Guinea, Nigeria, Sierra Leone, Gabon, Congo-Brazzaville), 12 countries where 3 are high income countries, 1 is upper middle income country, 5 are lower middle income countries, and 3 are low income countries. Perspectives came from research staff and study investigators. Focal populations are pregnant and lactating women. | Serious concerns: 4 papers contributed to review finding with relatively thin data | Low confidence | No or very minor concerns on coherence, no or very minor concerns on relevance, moderate concerns on methodological limitations (recruitment/data collection, analysis, link from data to findings, ethics, reflexivity), serious concerns on adequacy (4 papers contributed with relatively thin data) |
