# Supplementary material for: Factors influencing the participation of pregnant and lactating women in clinical trials: A mixed-methods systematic review
Source: PLoS Med. 2024 May 30;21(5):e1004405. doi: 10.1371/journal.pmed.1004405 (PMC11139290; doi:10.1371/journal.pmed.1004405)
Supplement: S5 Appendix — (DOCX) [file pmed.1004405.s005.docx]

S5. Appendix: Summaries of Quantitative Findings

| **Findings** | **Summary of Quantitative review findings** | **Contributing quantitative studies** | **Quality ratings** |
| --- | --- | --- | --- |
|  | **Interplay between perceived risks and benefits of participation in women’s decision-making** | | |
| **1** | **Women have a limited appetite for risk during pregnancy or lactation**  Quantitative evidence supported the qualitative findings that women were apprehensive about taking an experimental product medications as part of a trial during pregnancy or lactation primarily due to concerns of fetal or infant harm, side-effects, and the possibility of unknown longer-term negative sequelae. Prior knowledge of the health condition, information about drug safety in pregnant and non-pregnant populations, and information that large numbers of pregnant women had already enrolled in the trial were factors that increased willingness to participate. | [38, 41, 51, 67, 68, 71, 75, 77, 79, 80, 83, 94, 95] | 13 papers (3 high, 2 moderate, 7 low, 1 very low quality papers) |
| **2** | **Making trade-offs between risk and severity of condition and risk-benefit ratio of intervention**  Quantitative evidence supported the qualitative findings that, when coupled with risks that were considered minimal or manageable, women with greater knowledge about or direct exposure to the condition were more likely to participate in a vaccine or therapeutic trial. However, prior exposure to the medical condition did not consistently lead to higher participation in trials. | [51, 83, 94] | 3 papers (1 high, 2 low quality papers) |
| **3** | **Benefits to health arising from participation**  Quantitative evidence supported this finding that women were more willing to participate in a trial when they were convinced about the potential short and longer-term benefits of the intervention for the health of the fetus, and their own health and education. | [38, 41, 51, 75, 77, 80, 95] | 7 papers (2 high, 1 moderate, 3 low, 1 very low quality papers) |
| **4** | **Experiences and expectations of high-quality care motivate participation**  Quantitative evidence supported the qualitative finding that women expected trial participation to engender more and better-quality care through enhanced monitoring, more tests, better therapeutic treatment, and the general feeling of being provided a high standard of medical care. | [38, 41, 49, 51, 67, 68, 75, 80] | 8 papers (1 high, 2 moderate, 4 low, 1 very low quality papers) |
| **5** | **Knowledge of the rationale for study design features**  Quantitative evidence extended understanding of women’s views about participation in placebo-controlled trials. Some women expressed reluctance to participate due to the possibility of being assigned to the control or placebo group. However, others expressed that the uncertainty of assignment would not affect their decision, and for a minority, the possibility of assignment to the control condition motivated their participation as it could minimise risk but still provide ancillary benefits. Women were keen to be unblinded regarding the arm to which they were assigned, once the trial was complete. | [67, 77, 79, 80, 83] | 5 papers (2 high, 1 moderate, 2 low quality papers) |
| 6 | **Acceptability of the intervention is key to pregnant and lactating women's willingness to participate in a trial, and for research staff to recruit for a trial**  Quantitative evidence supported this qualitative finding that some women might be more willing to participate in a trial when they were less likely to be inconvenienced by trial procedures, additional and lengthy study visits. Decliners cited blood tests, additional scans, and availability of suitable non-invasive alternatives as reasons for non-participation. In the case of vaccine trials, quantitative data extended this qualitative finding by suggesting that women indicated greater acceptability of inactivated virus vaccines compared to live-attenuated virus vaccines. | [38, 41, 51, 80, 83] | 5 papers ( 2 high, 2 low, 1 very low quality papers) |
| 7 | **Fears around data sharing and use** [No quantitative evidence was identified in this domain] | [No quantitative evidence was identified in this domain] | [No quantitative evidence was identified in this domain] |
| 8 | **Altruistic motivations**  Quantitative evidence supported the qualitative finding that altruistic motivations influenced willingness to participate in trials, alongside personal benefits. Women expressed having a sense of fulfilment that participation would have a positive impact on women’s health in the future. | [38, 41, 49, 51, 67, 77, 80, 95] | 8 papers ( 2 high, 1 moderate, 4 low, 1 very low quality papers) |
| 9 | **Financial incentives**  Quantitative evidence extended this qualitative finding by suggesting that attitudes to financial compensation differed based on levels of education attainment. Less than one in 10 women discussed that financial incentives would increase their likelihood of participation in medication or vaccine-based research, whereas in another, four in 10 women agreed that they volunteered to participate due to financial compensation. | [41, 75, 97] | 3 papers (1 high, 1 moderate, 1 low quality papers) |
|  | **Engagement between women and the medical and research ecosystems** | | |
| 10 | **Roles of trust and power in the medical and research ecosystems**  Quantitative data supported the qualitative finding that trust (or lack thereof) in health workers, research teams and pharmaceutical companies affected participation. Some women felt pressured to participate by health workers and were disappointed by the lack of an individualised approach to recruitment. Among decliners of a vaccine trial, some noted that recommendations from a health worker could motivate a change of mind. | [38, 51, 75, 80, 95] | 5 papers ( 2 high, 1 moderate, 1 low, 1 very low quality papers) |
| 11 | **The role of therapeutic home and optimism**  [No quantitative evidence was identified in this domain] | [No quantitative evidence was identified in this domain] | [No quantitative evidence was identified in this domain] |
|  | **Gender norms and decision-making autonomy** | | |
| 12 | **Expectations of women’s roles as mothers and care givers**  [No quantitative evidence was identified in this domain] | [No quantitative evidence was identified in this domain] | [No quantitative evidence was identified in this domain] |
| 13 | **Role of bodily autonomy in decision-making**  Quantitative evidence supported qualitative findings that women believed in their capability to make decisions regarding trial participation, with some doing so autonomously and others receiving support from family members. | [38, 83] | 2 papers ( 1 high, 1 very low quality papers) |
| 14 | **Relationship dynamics, gender roles and norms are key to women’s attitudes to partner involvement and paternal consent**  [No quantitative evidence was identified in this domain] | [No quantitative evidence was identified in this domain] | [No quantitative evidence was identified in this domain] |
|  | **Factors affecting clinical trial recruitment** | | |
| 15 | **Developing trusting and reciprocal relationships with community as part of the research process**  [No quantitative evidence was identified in this domain] | [No quantitative evidence was identified in this domain] | [No quantitative evidence was identified in this domain] |
| 16 | **Increasing visibility and awareness of the trial**  Quantitative evidence extended the qualitative finding that women preferred to have information about trials through their health workers. | [67] | 1 paper with moderate quality paper |
| 17 | **Inadequate resources**  Quantitative evidence similarly reported that lack of infrastructure and limited time due to heavy workloads for health workers were barriers to including pregnant women in trials. | [50, 67, 88] | 3 papers (1 moderate, 2 low quality papers) |
| 18 | **Engaging health workers in trials**  Quantitative evidence supported qualitative findings that knowledge of the relevance, feasibility, and ethical obligations to include pregnant and lactating women in trials, perceptions that pregnant women were a vulnerable population, lack of interest in trials, and preferences for non-invasive treatment were factors influencing whether health workers encouraged pregnant women’s clinical trial participation. | [50, 52, 67, 88, 94, 95] | 6 papers (1 high, 1 moderate, 4 low quality papers) |
| 19 | **Research staff’s emotional orientations towards clinical trials**  [No quantitative evidence was identified in this domain] | [No quantitative evidence was identified in this domain] | [No quantitative evidence was identified in this domain] |
| 20 | **Women-centred approach encourages participation**  Quantitative data supported this qualitative finding of women noting the significance of having detailed and well explained trial information, including about risks and benefits, and adequate time to make decisions regarding participation. Some women expressed disappointment when they felt they had been ill-informed about study procedures by research staff. | [80, 95] | 2 papers with high quality papers |
| 21 | **Recruitment for intrapartum research**  Quantitative data extended this qualitative finding with most ethics committee members considering consent in-labour as ethical. Factors that ethics committee members considered when approving labour trials, included the level of risk involved and women’s ability to provide informed consent. Most ethics committee members also supported the involvement of partners in the consent process. Aligned with the qualitative data, women expressed a preference to be approached for a labour trial earlier to have adequate time for discussion and an informed decision. | [76, 79, 80] | 3 papers with 1 high and 2 low quality papers |
|  | **Upstream factors affecting the research ecosystem** | | |
| 22 | **Factors affecting motivation of study investigators**  [No quantitative evidence was identified in this domain] | [No quantitative evidence was identified in this domain] | [No quantitative evidence was identified in this domain] |
| 23 | **Challenges in gaining ethical approvals for trials with pregnant women**  Quantitative evidence supported qualitative findings that obtaining regulatory approval for clinical trials that include pregnant women was challenging due to ethics committees’ preference for observational studies over trials, and varied opinions on the inclusion of pregnant women and what constituted minimal risk. Most ethics committee members were also aware that they did not have adequate policy or guidance to inform their decisions to ensure equitable subject selection. | [76, 88, 93] | 3 papers with low quality papers |
| 24 | **Role of funders**  [No quantitative evidence was found identified in this domain] | [No quantitative evidence was identified in this domain] | [No quantitative evidence was identified in this domain] |
