# Supplementary material for: Factors influencing the participation of pregnant and lactating women in clinical trials: A mixed-methods systematic review
Source: PLoS Med. 2024 May 30;21(5):e1004405. doi: 10.1371/journal.pmed.1004405 (PMC11139290; doi:10.1371/journal.pmed.1004405)
Supplement: S6 Appendix — (DOCX) [file pmed.1004405.s006.docx]

S6. Appendix: Characteristics of Included Papers

| **Author** | **Title** | **Country** | **Designs** | **Participants** | **Sample size** | **Focal population** | **Type of intervention** | **Trial area** |
| --- | --- | --- | --- | --- | --- | --- | --- | --- |
| Atal 2018 | Assessment of the understanding of informed consent including participants' experiences, and generation of a supplemental consent decision aid for Gestational Diabetes Mellitus (GDM) research. | Republic of Ireland | Quantitative (self-completed survey, observation) | Pregnant women | 15 | Pregnant women | Therapy | Complications of pregnancy/childbirth (Gestational diabetes melitus) |
| Baker 2005 | Factors that influence women's decisions about whether to participate in research: an exploratory study | United  Kingdom | Qualitative (in-person interviews, group discussion) | Postpartum women^a^ | 17 | Pregnant women | Not specified | Not specified |
| Ballantyne 2017 | The experiences of pregnant women in an interventional clinical trial: Research In Pregnancy Ethics (RIPE) study | New Zealand | Qualitative (in-person interview) | Postpartum women^a^ | 20 | Pregnant women | Therapy | Complications of pregnancy/childbirth (Gestational diabetes mellitus) and Infectious disease (Group B streptococcus) |
| Bevan 2023 | A questionnaire-based study exploring participant perspectives in a perinatal human challenge trial | United Kingdom | Quantitative (self-completed survey) | Postpartum women^a^ | - Pre-participation: 27  - Post participation: 15 | Pregnant women | Therapy | Infectious disease and Fetal/newborn (mother-to-infant  upper respiratory commensal transmission) |
| Brandon 2011 | Ethical barriers to perinatal mental health research and evidence-based treatment: an empirical study | United States | Qualitative (in-person and phone interviews) | Study investigators | 14 | Pregnant and lactating women | Not specified | Mental health (Perinatal mental health) |
| Brandon 2014 | Ethical challenges in designing, conducting, and reporting research to improve the mental health of pregnant women: the voices of investigators and IRB members | United States | Qualitative (phone interviews) | - Study investigators  - Ethics committee members | - Study investigators: 15  - Ethics committee members: 6 | Pregnant and lactating women | Not specified | Mental health (Perinatal mental health) |
| Compaore 2018 | Fear and rumours regarding placental biopsies in a malaria-in-pregnancy trial in Benin | Benin, The Gambia and Burkina Faso | Qualitative (interviews, group discussion, observation) | - Postpartum women^a^  - Partners of postpartum women  - Community leaders/members(Village chief, traditional healers)  - Research staff (Research team members, community health workers)  - Health workers (Midwives) | - Postpartum women: 25  - Partners: 3  - Village chiefs: 11  - Traditional healers: 4  - Community health workers: 19  - Research team members: 7  - Midwives: 1 | Pregnant women | Therapy | Infectious disease (Malaria) |
| Corneli 2007 | Involving communities in the design of clinical trial protocols: The BAN Study in Lilongwe, Malawi | Malawi | Qualitative (interviews, group discussion) | - Postpartum women^a^  - Pregnant women  - Family members of postpartum women  - Partners of postpartum women  - Community leaders | - Postpartum women: 23  - Pregnant women: 25  - Family members of postpartum women: 26  - Partners of postpartum women: 26  - Community leaders 7 | Pregnant and lactating women | Therapy | Infectious disease and Fetal/newborn (HIV/AIDS) |
| Coulibaly-Traore 2003^b^ | [The Ditrame (ANRS 049) clinical trial aimed at reducing the mother-child transmission of HIV in Abidjan. Participants' understanding of the trial principles] | Cote D'Ivoire | Qualitative (interview survey) | Postpartum women^a^ | 57 | Lactating women | Therapy | Infectious disease and Fetal/newborn (HIV/AIDS) |
| Dorey 2023 | Qualitative interview study exploring the perspectives of pregnant women on participating in controlled human infection research in the UK | United Kingdom | Qualitative (in-person interviews) | Pregnant women | 12 | Pregnant women | Therapy | Infectious disease and Fetal/newborn (mother-to-infant  upper respiratory commensal transmission) |
| Hollander 2018 | Power difference and risk perception: mapping vulnerability within the decision process of pregnant women towards clinical trial participation in an urban middle-income setting | Ghana | Qualitative (in-person interviews, group discussion) | - Pregnant women  - Health workers (Midwives) | - Pregnant women: 17  - Midwives: 2 | Pregnant women | Therapy | Complications of pregnancy/childbirth (Hypertensive disorders of pregnancy) |
| Ferguson 2000 | Testing a drug during labour: the experiences of women who participated in a clinical trial | United Kingdom | Mixed-methods (in-person and phone interviews) | Postpartum women^a^ | 104 | Pregnant women | Therapy | Complications of pregnancy/childbirth (Pain relief during labour) |
| Gagneux-Brunon 2022 | Midwives’ attitudes toward participation of pregnant individuals in a  preventive vaccine hypothetical clinical trial | France | Quantitative (Self-completed survey) | Health workers (Midwives, midwifery students) | - Midwives: 256  - Midwifery students: 142 | Pregnant women | Vaccine | Infectious disease (Respiratory syncytial virus) |
| Goldfarb 2018 | Pregnant women’s attitudes toward zika virus vaccine trial participation | United States | Quantitative (Self-completed survey) | - Pregnant women  - Postpartum women^a^ | 128 | Pregnant and lactating women | Vaccine | Infectious disease (Zika virus) |
| Haas 2010 | Women’s health care providers’ attitudes toward research in pregnancy | United States | Quantitative (Self-completed survey) | Health workers (Obstetric physicians, nurses,  ancillary medical providers, staff members in maternity care) | - Obstetric physicians: 16  - Nurses: 82  - Ancillary medical providers, staff members in maternity care: 33 | Pregnant women | Not specified | Not specified |
| Hallowell 2016 | The role of therapeutic optimism in recruitment to a clinical trial in a peripartum setting: balancing hope and uncertainty | United Kingdom | Qualitative (Phone interviews) | - Health workers (Clinical midwives, obstetricians)  - Research staff (Research midwives) | - Clinical midwives: 6  - Obstetricians: 10  - Research midwives:11 | Pregnant women | Therapy | Complications of pregnancy/childbirth (Treatment of retained placenta) |
| Hanrahan 2022 | Theory-guided interviews identified behavioral barriers and enablers to healthcare professionals recruiting participants to maternity trials | Republic of Ireland, United Kingdom | Qualitative (Phone and online interviews) | - Health workers (Clinical midwives/nurses, doctor)  - Research staff (Research midwives)  - Study investigators | - Clinical midwives/nurses: 4  - Doctor: 2  - Research midwives:11  - Study investigators: 5 | Pregnant and lactating women | Not specified | Complications of pregnancy/childbirth (Maternity care) |
| Harrington 2017 | Recruitment barriers for prophylactic vaccine trials: a study in Belgium | Belgium | Mixed-methods (Self-completed survey, online interviews) | - Study investigators  - Research staff (clinical research organisation)  - Funders (sponsor organisation)  - Ethics committee members | - Study investigators: 10  - Clinical research organisation: 7  - Sponsor organisation:5  - Ethics committee members: 3 | Pregnant women | Vaccine | Not specified |
| Houghton 2018 | What women think about consent to research at the time of an obstetric emergency: a qualitative study of the views of a cohort of World Maternal Antifibrinolytic Trial participants | United Kingdom | Qualitative (In-person interviews) | Postpartum women^a^ | 15 | Pregnant women | Therapy | Complications of pregnancy/childbirth (Postpartum haemorrhage) |
| Jaffe 2020 | Pregnant women’s perceptions of risks and benefits when considering participation in vaccine trials | United States | Qualitative (In-person interviews) | - Pregnant women  - Postpartum women^a^ | 13 | Pregnant women | Vaccine | Infectious disease (Zika virus) |
| Karafillakis 2021 | 'My primary purpose is to protect the unborn child': understanding pregnant women's perceptions of maternal vaccination and vaccine trials in Europe. | France, Germany, Italy, Spain and United Kingdom | Qualitative (Interviews, group discussion) | Pregnant women | 258 | Pregnant women | Vaccine | Not specified |
| Kenyon 2006 | Participating in a trial in a critical situation: a qualitative  study in pregnancy | United Kingdom | Qualitative (In-person interviews) | Postpartum women^a^ | 20 | Pregnant women | Therapy | Complications of pregnancy/childbirth (Preterm labour) |
| Kislovskiy 2022 | Motives and risk perceptions of participants in a phase 1 trial for Hepatitis C Virus investigational therapy in pregnancy | United States | Qualitative (In-person interviews) | Pregnant women | 9 | Pregnant women | Therapy | Infectious disease and Fetal/newborn (Hepatitis C) |
| Lawton 2016 | Recruiting and consenting into a peripartum trial in an emergency setting: a qualitative study of the experiences and views of women and healthcare professionals | United Kingdom | Qualitative (In-person and phone interviews) | - Pregnant women  - Health workers (Obstetricians, midwifery staff)  - Research staff (Research midwives) | - Pregnant women  : 22  - Health and research personnel: 27 | Pregnant women | Therapy | Complications of pregnancy/childbirth (Postpartum haemorrhage) |
| Lie 2022 | Emesis in pregnancy - a qualitative study on trial recruitment failure from the EMPOWER internal pilot | United Kingdom | Qualitative (Phone and email interviews) | - Postpartum women^a^  - Study investigators  - Research staff (Research midwives/ nurses) | - Postpartum women: 21  - Study investigators: 6  - Research midwives/nurses: 16 | Pregnant women | Therapy | Complications of pregnancy/childbirth (Severe emesis in pregnancy) |
| Lyerly 2012 | Women’s views about participating in research while pregnant | United States | Qualitative (Interviews) | Postpartum women^a^ | 22 | Pregnant women | Vaccine | Infectious disease (H1N1 vaccine trials) |
| Marban-Castro 2021 | Acceptability of clinical trials on Covid-19 during pregnancy among pregnant women and healthcare providers: a qualitative study | Spain | Qualitative (Phone and online interviews, field notes) | - Pregnant women  - Health workers (Doctors, nurses) | - Pregnant women: 24  - Doctors and nurses: 6 | Pregnant women | Vaccine | Infectious disease (Covid-19) |
| MartinezPerez 2018 | ‘Researchers have love for life’:  opportunities and barriers to engage pregnant  women in malaria research in post‑Ebola Liberia | Liberia | Qualitative (Interviews, group discussion) | - Pregnant women  - Health workers (Medical, laboratory and management staff)  - Community leaders | - Pregnant women: 17  - Medical, laboratory and management staff: 10  - Community leaders: 11 | Pregnant women | Not specified | Infectious disease (Malaria) |
| Mastroianni 2020 | The pathway forward: insights on factors that facilitate research with pregnant women. | United States | Qualitative (In-person, phone and online interviews) | - Study investigators  - Ethics committee members (ethics committee members, University administrators) | - Study investigators: 9  - Ethics committee members and University administrators: 5 | Pregnant women | Therapy | Not specified |
| McQuaid 2016 | Factors influencing women's attitudes towards antenatal vaccines, group B Streptococcus and clinical trial participation in pregnancy: an online survey. | United Kingdom | Quantitative (Self-completed survey) | Reproductive aged women | 1,013 | Pregnant women | Vaccine | Infectious disease (Group B streptococcus) |
| McQuaid 2018 | Antenatal vaccination against Group B streptococcus: attitudes of pregnant women and healthcare professionals in the UK towards participation in clinical trials and routine implementation. | United Kingdom | Quantitative (Self-completed survey) | - Pregnant women  - Health workers (Maternity professionals, neonatal doctors) | - Pregnant women: 269  - Maternity professionals:273  - Neonatal doctors: 97 | Pregnant women | Vaccine | Infectious disease (Group B streptococcus) |
| Mohanna 1999 | Withholding consent to participate in clinical trials:  decisions of pregnant women | United Kingdom | Qualitative (In-person interviews) | Postpartum women^a^ | 18 | Pregnant women | Therapy | Complications of pregnancy/childbirth (Preterm labour) |
| Monteiro 2019 | Reasons given by pregnant women for participating in a clinical trial aimed at preventing premature delivery: a qualitative analysis | Brazil | Qualitative (Interview survey) | Postpartum women^a^ | 208 | Pregnant women | Therapy | Complications of pregnancy/childbirth (Preterm birth) |
| Myles 2018 | A multicenter investigation of factors influencing women’s participation in clinical trials | United States | Quantitative (Self-completed survey) | - Pregnant women  - Postpartum women^a^ | 3,773 | Pregnant and lactating women | Not specified | Not specified |
| Nikcevic 2019 | Reasons for accepting or declining participation in the ASPRE trial: a qualitative study with women at high risk of preterm pre‐eclampsia | United Kingdom | Qualitative (In-person interviews) | Pregnant women | 27 | Pregnant women | Therapy | Complications of pregnancy/childbirth (Pre-eclampsia) |
| Osarfo 2020 | Participation of Ghanaian pregnant women in an antimalarial drug trial: willingness, experiences and perceptions | Ghana | Qualitative (Interviews) | - Pregnant women  - Partners of pregnant women | - Pregnant women: 20  - Partners of pregnant women: 5 | Pregnant women | Therapy | Infectious disease (Malaria) |
| OudeRengerink 2015 | Pregnant womens’ concerns when invited to a randomized trial: a qualitative case control study | Netherlands | Qualitative (In-person and phone interviews) | - Pregnant women  - Postpartum women^a^ | 21 | Pregnant women | Not specified | Not specified |
| Palmer 2016 | Optimizing participation of pregnant women  In clinical trials: factors influencing decisions about participation in medication and vaccine trials | Canada | Quantitative (Self-completed survey) | Pregnant women | 110 | Pregnant women | Therapy | Not specified |
| Reid 2011 | The ethics of obtaining consent in labour for research | Australia | Quantitative (Self-completed survey) | Ethics committee members | 138 | Pregnant women | Not specified | Not specified |
| Rodger 2003 | Participation of pregnant women in clinical trials: will they participate and why? | Canada | Quantitative (In-person interview, interview survey) | Pregnant women | 71 | Pregnant women | Therapy | Complications of pregnancy/childbirth (Complications during pregnancy in thrombophilic women) |
| Salami 2022 | The promise, problems, and pitfalls of including  pregnant women in clinical trials of Lassa fever  vaccine: a qualitative assessment of sub-Sahara Africa  investigators’ perception | Benin, Burkina Faso, Ghana, Mali, Guinea, Nigeria, Sierra Leone, Gabon, and Congo-Brazzaville | Qualitative (In-person and phone interviews) | Study investigators | 6 | Pregnant women | Vaccine | Infectious disease (Lassa fever) |
| Scott 2023 | Motivations and demographic differences in pregnant individuals  in the decision to participate in research | United States | Quantitative (self-completed survey) | Postpartum women^a^ | 400 | Pregnant women | Therapy | Complications of pregnancy/childbirth (Pain relief after vaginal birth) |
| Smyth 2009 | Women’s experiences of participating in the  Magpie trial: a postal survey in the  United kingdom | United Kingdom | Quantitative (Self-completed survey) | Postpartum women^a^ | 619 | Pregnant women | Therapy | Complications of pregnancy/childbirth (Eclampsia) |
| Smyth 2012 | Deciding to join a perinatal randomised controlled trial: experiences and views of pregnant women enroled in the Magpie trial | United Kingdom | Qualitative (Interviews) | Postpartum women^a^ | 40 | Pregnant women | Therapy | Complications of pregnancy/childbirth (Eclampsia) |
| Snowdon 2012 | Views of emergency research (VERA): A qualitative study of women and their partners’ views of recruitment to trials in severe postpartum haemorrhage | United Kingdom | Qualitative (Interviews) | - Postpartum women^a^  - Partners of postpartum women | - Postpartum women:9  - Partners of postpartum women: 6 | Pregnant women | Therapy | Complications of pregnancy/childbirth (Postpartum haemorrhage) |
| Strommer 2018 | Improving recruitment to clinical trials during pregnancy: a mixed methods investigation | United Kingdom | Mixed-methods (Self-completed survey, In-person and phone interviews) | Postpartum women^a^ | 30 | Pregnant women | Therapy | Non-communicable disease (Osteoporosis) |
| Sullivan 2018 | Women’s views about a paternal consent requirement for biomedical research in pregnancy | United States, Malawi | Qualitative (Interviews) | - Pregnant women  - Postpartum women^a^ | 140 | Pregnant women | Therapy | Infectious disease and Fetal/newborn (HIV/AIDS) |
| Sullivan 2020 | Views among Malawian women about joining HIV prevention clinical trials when pregnant | Malawi | Qualitative (Interviews) | Postpartum women^a^ | 35 | Pregnant women | Therapy | Infectious disease (HIV/AIDS) |
| Sweeney 2022 | Deferred consent in emergency obstetric research: findings from qualitative interviews with women and recruiters in the ACROBAT pilot trial for severe postpartum haemorrhage | United Kingdom | Qualitative (In-person interviews) | - Postpartum women^a^  - Research staff (Research midwives, clinical trials practitioners, research team) | - Postpartum women: 10  - Research staff: 4 | Pregnant women | Therapy | Complications of pregnancy/childbirth (Postpartum haemorrhage) |
| Sweet 2023 | Pregnant women with diabetes and their clinician's  experience of participating in a pilot randomised controlled  trial of corticosteroid administration in late pregnancy:  A qualitative study | Australia | Qualitative (In-person and phone interviews) | - Postpartum women^a^  - Health workers (Endocrinology staff, clinical midwives)  - Research staff (Research midwives) | - Postpartum women: 13  - Health workers and research staff: 9 | Pregnant women | Therapy | Complications of pregnancy/childbirth (Respiratory distress syndrome in newborns due to gestational or pregestational diabetes) |
| Trahan 2021 | Physician perspectives on including pregnant women in Covid-19 clinical trials: time for a paradigm change | Canada | Quantitative (Self-completed survey) | Study investigators | 168 | Pregnant women | Therapy | Infectious disease (Covid-19) |
| vanderZande 2017 | A qualitative study on acceptable levels of risk for pregnant women in clinical research | Netherlands | Qualitative (Interviews, group discussion) | - Pregnant women  - Ethics committee members  - Regulators | - Pregnant women: 14  - Ethics committee members and regulators: 26 | Pregnant women | Therapy | Complications of pregnancy/childbirth (Preterm birth) |
| vanderZande 2019 | A qualitative study on stakeholders’ views on the participation of pregnant women in the APOSTEL VI study: a low-risk obstetrical RCT | Netherlands | Qualitative (Interviews, group discussion) | - Pregnant women  - Ethics committee members  - Regulators  - Research staff (Research midwives)  - Health workers (Gynaecologist, gynaecologist-in-training) | - Pregnant women: 14  - Ethics committee members: 5  - Regulators: 7  - Research midwives: 5  - Gynaecologist and Gynaecologist-in-training: 9 | Pregnant women | Therapy | Complications of pregnancy/childbirth (Preterm birth) |
| Wada 2018 | Clinical research with pregnant women: perspectives of pregnant women, health care providers, and researchers | Canada | Qualitative (Interviews) | - Pregnant women  - Health workers  - Study investigators | - Pregnant women: 12  - Health workers: 10  - Study investigators: 9 | Pregnant women | Not specified | Not specified |
| Wallace 2021 | Participation in a randomised controlled trial (RCT) of metformin in gestational diabetes mellitus (GDM): pregnant women’s perceptions and experiences of the decision-making process | Republic of Ireland, United Kingdom | Qualitative (In-person interviews) | Postpartum women^a^ | 11 | Pregnant women | Therapy | Complications of pregnancy/childbirth (Gestational diabetes melitus) |
| White 2021 | IRB decision-making about minimal risk research with pregnant participants | United States | Quantitative (Self-completed survey) | Ethics committee members (Ethics committee members, administrators) | - Ethics committee members: 93  - Administrators: 39 | Pregnant women | Therapy | Infectious disease (HIV/AIDS) |
| Wilcox 2019 | Attitudes of pregnant women and healthcare professionals toward clinical trials and routine implementation of antenatal vaccination against respiratory syncytial virus: a multicenter questionnaire study | United Kingdom | Quantitative (Self-completed survey) | - Pregnant women  - Health workers (obstetricians, midwives, others (not specified)) | - Pregnant women: 321  - Health workers: 204 | Pregnant women | Vaccine | Fetal/newborn (Respiratory syncytial virus) |
| Xu 2023 | Peripartum women’s perspectives on research study participation in the OneFlorida Clinical Research Consortium during COVID-19 pandemic | United States | Quantitative (Self-completed survey) | - Pregnant women  - Postpartum women^a^ | 533 | Pregnant and lactating women | Not specified | Infectious disease (Covid-19) |
| Zhao 2018 | Factors infuencing the recruitment of lactating women in a clinical trial involving direct oral anticoagulants: a qualitative study | United Kingdom | Qualitative (Group discussion) | - Lactating women  - Women who had breastfeed within past 5 years | 8 | Lactating women | Therapy | Non-communicable disease (Venous thromboembolism) |
| Zhao 2021 | Clinical drug trial participation: perspectives of pregnant women and their spouses | China | Quantitative (Self-completed survey) | - Pregnant women  - Partners of pregnant women | - Pregnant women: 115  - Partners of pregnant women: 91 | Pregnant women | Therapy | Not specified |

^a^: Women who participated/ did not participate in a trial during pregnancy and participated in observational studies during postpartum for their experiences participating/not participating in trial

^b^: a study published in French
