# Supplementary material for: Factors influencing the participation of pregnant and lactating women in clinical trials: A mixed-methods systematic review
Source: PLoS Med. 2024 May 30;21(5):e1004405. doi: 10.1371/journal.pmed.1004405 (PMC11139290; doi:10.1371/journal.pmed.1004405)
Supplement: S7 Appendix — (DOCX) [file pmed.1004405.s007.docx]

S7 Appendix: Critical Appraisal

Critical appraisal of qualitative studies

| Study details | Screening questions | | 1. QUALITATIVE STUDIES QUESTIONS | | | | | | | MMAT RATING |
| --- | --- | --- | --- | --- | --- | --- | --- | --- | --- | --- |
| Author | S1. Are there clear research questions? | S2. Do the collected data allow to address the research questions? | 1.1. Is the qualitative approach appropriate to answer the research question? (Aim, appropriateness of a qualitative approach) | 1.2. Are the qualitative data collection methods adequate to address the research question? (recruitment, data collection) | 1.3. Are the findings adequately derived from the data? (rigor in analysis) | 1.4. Is the interpretation of results sufficiently substantiated by data? (link from data to findings) | 1.5. Is there coherence between qualitative data sources, collection, analysis and interpretation? (overall design from start to finish) | 1.6. Have ethical issues been taken into consideration? (consent, confidentiality, ethics approval) | 1.7. Is relationship between researcher and participants adequately considered? (interaction and reflection on how research team influences design & implementation) |  |
| Baker 2005 | Yes | Yes | Yes | Partial | Partial | Yes | Yes | Partial | No | “Moderate” (minor flaws impacting credibility/validity) |
| Ballantyne 2017 | Yes | Yes | Yes | Partial | Yes | Yes | Yes | Partial | No | “Moderate” (minor flaws impacting credibility/validity) |
| Brandon 2011 | Yes | Yes | Yes | Yes | Unclear | Partial | Partial | Partial | No | “Very low” (significant flaws impacting credibility/validity) |
| Brandon 2014 | Yes | Yes | Yes | Yes | Yes | Yes | Yes | Partial | No | “High” (no significant flaws) |
| Compaore 2018 | Yes | Yes | Yes | Partial | Unclear | Yes | Partial | Partial | No | “Low” (some flaws likely to impact credibility/validity) |
| Corneli 2007 | Yes | Yes | Yes | Yes | Yes | Yes | Yes | Yes | Yes | “High” (no significant flaws) |
| Coulibaly-Traore 2003 | Yes | Yes | Yes | Partial | Unclear | Yes | Partial | Partial | No | “Very low” (significant flaws impacting credibility/validity) |
| Dorey 2023 | Yes | Yes | Yes | Yes | Yes | Yes | Yes | Yes | No | “High” (no significant flaws) |
| Hollander 2018 | Yes | Yes | Yes | Partial | Partial | Partial | Partial | Partial | No | “Very low” (significant flaws impacting credibility/validity) |
| Hallowell 2016 | Yes | Yes | Yes | Yes | Yes | Yes | Yes | Partial | No | “High” (no significant flaws) |
| Hanrahan 2022 | Yes | Yes | Yes | Yes | Yes | Yes | Yes | Yes | Yes | “High” (no significant flaws) |
| Houghton 2018 | Yes | Yes | Yes | Yes | Yes | Yes | Yes | Partial | Partial | “High” (no significant flaws) |
| Jaffe 2020 | Yes | Yes | Yes | Yes | Partial | Yes | Yes | Yes | No | “High” (no significant flaws)” |
| Karafillakis 2021 | Yes | Yes | Yes | Yes | Yes | Yes | Yes | Yes | No | “High” (no significant flaws)” |
| Kenyon 2006 | Yes | Yes | Yes | Yes | Yes | Yes | Yes | Partial | Partial | “High” (no significant flaws)” |
| Kislovskiy 2022 | Yes | Yes | Yes | Yes | Yes | Yes | Yes | Partial | No | “High” (no significant flaws)” |
| Lawton 2016 | Yes | Yes | Yes | Yes | Yes | Yes | Yes | Yes | No | “High” (no significant flaws)” |
| Lie 2022 | Yes | Yes | Yes | Partial | Yes | Yes | Yes | Yes | Partial | “Moderate” (minor flaws impacting credibility/validity) |
| Lyerly 2012 | Yes | Yes | Yes | Partial | Yes | Yes | Yes | Partial | No | “Moderate” (minor flaws impacting credibility/validity) |
| Marbán-Castro 2021 | Yes | Yes | Yes | Partial | Partial | Yes | Partial | Yes | No | “Low” (some flaws likely to impact credibility/validity) |
| Martinez 2018 | Yes | Yes | Yes | Partial | Yes | Yes | Yes | Yes | Yes | “High” (no significant flaws) |
| Mastroianni 2020 | Yes | Yes | Yes | Yes | Partial | Yes | Yes | Yes | No | “Moderate” (minor flaws impacting credibility/validity) |
| Mohanna 1999 | Yes | Yes | Yes | Yes | Yes | Yes | Yes | Partial | No | “High” (no significant flaws) |
| Monteiro 2019 | Yes | Yes | Yes | Partial | Yes | Yes | Partial | Partial | No | “Moderate” (minor flaws impacting credibility/validity) |
| Nikcevic 2019 | Yes | Yes | Yes | Yes | Yes | Yes | Yes | Partial | No | “High” (no significant flaws) |
| Osarfo 2020 | Yes | Yes | Yes | Partial | Yes | Yes | Yes | Partial | No | “Moderate” (minor flaws impacting credibility/validity) |
| OudeRengerink 2015 | Yes | Yes | Yes | Yes | Yes | Yes | Yes | Yes | No | “High” (no significant flaws) |
| Salami 2022 | Yes | Yes | Yes | Partial | Yes | Partial | Yes | Partial | No | “Low” (some flaws likely to impact credibility/validity) |
| Smyth 2012 | Yes | Yes | Yes | Yes | Yes | Yes | Yes | Partial | No | “High” (no significant flaws) |
| Snowdon 2012 | Yes | Yes | Yes | Yes | Yes | Yes | Yes | Yes | Partial | “High” (no significant flaws) |
| Sullivan 2018 | Yes | Yes | Yes | Partial | Yes | Yes | Yes | Partial | No | “High” (no significant flaws) |
| Sullivan 2020 | Yes | Yes | Yes | Yes | Yes | Yes | Yes | Partial | No | “High” (no significant flaws) |
| Sweeney 2022 | Yes | Yes | Yes | Yes | Yes | Partial | Yes | Yes | No | “Moderate” (minor flaws impacting credibility/validity) |
| Sweet 2023 | Yes | Yes | Yes | Partial | Partial | Yes | Partial | Yes | No | “Low” (some flaws likely to impact credibility/validity) |
| vanderZande 2017 | Yes | Yes | Yes | Partial | Yes | Yes | Yes | Partial | No | “High” (no significant flaws) |
| vanderZande 2019 | Yes | Yes | Yes | Partial | Yes | Yes | Yes | Partial | No | “High” (no significant flaws) |
| Wada 2018 | Yes | Yes | Yes | Yes | Yes | Yes | Yes | Partial | Partial | “High” (no significant flaws) |
| Wallace 2021 | Yes | Yes | Yes | Yes | Yes | Yes | Yes | Yes | Yes | “High” (no significant flaws) |
| Zhao 2018 | Yes | Yes | Yes | Yes | Yes | Yes | Yes | Yes | No | “High” (no significant flaws) |

Critical appraisal of quantitative studies

| Study details | Screening questions | | 4. QUANTITATIVE DESCRIPTIVE STUDIES QUESTIONS | | | | | | MMAT RATING |
| --- | --- | --- | --- | --- | --- | --- | --- | --- | --- |
| Author | S1. Are there clear research questions? | S2. Do the collected data allow to address the research questions? | 4.1. Is the sampling strategy relevant to address the research question? | 4.2. Is the sample representative of the target population? | 4.3. Are the measurements appropriate? | 4.4. Is the risk of nonresponse bias low? | 4.5. Have ethical issues been taken into consideration? (consent, confidentiality, ethics approval) | 4.6. Is the statistical analysis appropriate to answer the research question? |  |
| Atal 2018 | Yes | Yes | Yes | Unclear | Yes | Unclear | Partial | Unclear | “Very low” (significant flaws impacting credibility/validity) |
| Bevan 2023 | Yes | Yes | Yes | No | Partial | No | Yes | Yes | “Low” (some flaws likely to impact credibility/validity) |
| Gagneux-Brunon qua2022 | Yes | Yes | Yes | Yes | Yes | No | Partial | Partial | “Low” (some flaws likely to impact credibility/validity) |
| Goldfarb 2018 | Yes | Yes | Partial | No | Yes | Unclear | Partial | Yes | “Low” (some flaws likely to impact credibility/validity) |
| Haas 2010 | Yes | Yes | Yes | Unclear | Yes | No | Partial | Yes | “Low” (some flaws likely to impact credibility/validity) |
| McQuaid 2016 | Yes | Yes | Yes | Yes | Partial | Yes | Unclear | Yes | “Low” (some flaws likely to impact credibility/validity) |
| McQuaid 2018 | Yes | Yes | Partial | Unclear | Yes | Yes | Partial | Yes | “Moderate” (minor flaws impacting credibility/validity) |
| Myles 2018 | Yes | Yes | Unclear | Unclear | Yes | Partial | Partial | Yes | “Low” (some flaws likely to impact credibility/validity) |
| Palmer 2016 | Yes | Yes | Partial | Unclear | Yes | Yes | Partial | Yes | “Moderate” (minor flaws impacting credibility/validity) |
| Reid 2011 | Yes | Yes | Yes | Unclear | Unclear | Partial | Partial | Partial | “Low” (some flaws likely to impact credibility/validity) |
| Rodger 2003 | Yes | Yes | Partial | Unclear | Yes | Partial | Partial | Yes | “Low” (some flaws likely to impact credibility/validity) |
| Scott 2023 | Yes | Yes | Yes | No | Unclear | Partial | Partial | Yes | “Low” (some flaws likely to impact credibility/validity) |
| Smyth 2009 | Yes | Yes | Yes | Partial | Yes | Yes | Partial | Yes | "High” (no significant flaws) |
| Trahan 2021 | Yes | Yes | Yes | Unclear | Yes | Unclear | Partial | Yes | “Low” (some flaws likely to impact credibility/validity) |
| White 2021 | Yes | Yes | Yes | Unclear | Yes | Unclear | Partial | Yes | “Low” (some flaws likely to impact credibility/validity) |
| Wilcox 2019 | Yes | Yes | Yes | Unclear | Partial | Unclear | Partial | Yes | “Low” (some flaws likely to impact credibility/validity) |
| Xu 2023 | Yes | Yes | Yes | Unclear | Yes | Yes | Partial | Yes | "High” (no significant flaws) |
| Zhao 2021 | Yes | Yes | Yes | Yes | Yes | Yes | Partial | Yes | "High” (no significant flaws) |

Critical appraisal of mixed methods studies

| Study details | Author | Ferguson 2000 | Harrington 2017 | Strommer 2018 |
| --- | --- | --- | --- | --- |
| Screening questions | S1. Are there clear research questions? | Yes | Yes | Yes |
|  | S2. Do the collected data allow to address the research questions? | Yes | Yes | Yes |
| 1. Qualitative studies questions | 1.1. Is the qualitative approach appropriate to answer the research question? (Aim, appropriateness of a qualitative approach) | Yes | Yes | Yes |
|  | 1.2. Are the qualitative data collection methods adequate to address the research question? (recruitment, data collection) | Unclear | Yes | Yes |
|  | 1.3. Are the findings adequately derived from the data? (rigor in analysis | Unclear | Unclear | Yes |
|  | 1.4. Is the interpretation of results sufficiently substantiated by data? (link from data to findings) | Yes | No | Yes |
|  | 1.5. Is there coherence between qualitative data sources, collection, analysis and interpretation? (overall design from start to finish) | Partial | Partial | Yes |
|  | 1.6. Have ethical issues been taken into consideration? (consent, confidentiality, ethics approval) | Partial | Unclear | Partial |
|  | 1.7. Is relationship between researcher and participants adequately considered? (interaction and reflection on how research team influences design & implementation) | No | No | No |
| 4. Quantitative descriptive studies questions | 4.1. Is the sampling strategy relevant to address the research question? | Unclear | Yes | Yes |
|  | 4.2. Is the sample representative of the target population? | Unclear | Unclear | Unclear |
|  | 4.3. Are the measurements appropriate? | Yes | Yes | Yes |
|  | 4.4. Is the risk of nonresponse bias low? | No | No | Yes |
|  | 4.5. Have ethical issues been taken into consideration? (consent, confidentiality, ethics approval) | Partial | Unclear | Partial |
|  | 4.6. Is the statistical analysis appropriate to answer the research question? | Yes | Yes | Yes |
| 5. Mixed methods studies questions | 5.1. Is there an adequate rationale for using a mixed methods design to address the research question? | Yes | Unclear | Yes |
|  | 5.2. Are the different components of the study effectively integrated to answer the research question? | Yes | Yes | Yes |
|  | 5.3. Are the outputs of the integration of qualitative and quantitative components adequately interpreted? | Yes | Partial | Yes |
|  | 5.4. Are divergences and inconsistencies between quantitative and qualitative results adequately addressed? | Yes | Unclear | Unclear |
|  | 5.5. Do the different components of the study adhere to the quality criteria of each tradition of the methods involved? | Partial | Partial | Partial |
| MMAT RATING | | “Low” (some flaws likely to impact credibility/validity) | “Very low” (significant flaws impacting credibility/validity) | "High” (no significant flaws) |
