# Supplementary material for: Factors influencing the participation of pregnant and lactating women in clinical trials: A mixed-methods systematic review
Source: PLoS Med. 2024 May 30;21(5):e1004405. doi: 10.1371/journal.pmed.1004405 (PMC11139290; doi:10.1371/journal.pmed.1004405)
Supplement: Attachment — Submitted filename: shankar.pdf [file pmed.1004405.s008.pdf]

This paper describes a review of studies of barriers and facilitators to participation by women in clinical trials.

I was asked for a statistical report and I interpret that to include all aspects of the design and conduct of the study.

Points of detail

Page 4 I can understand most of the exclusions but why exclude dissertations and theses? I suppose one could argue that high–quality ones will have, at least in part, been published but I doubt that is universal.

Page 5 Why was substance use excluded? We ought to know the reason.

Page 5 Good to see a specialist in information involved, a neglected source of expertise.

Page 5 I am surprised that the authors used a generic AI system rather than a specialist machine translation service like DeepL or Google Translate. There is some limited empirical work on using Google Translate in reviews (Jackson et al., 2019). Having the outcome checked by a human is clearly a good idea.

Page 6 It might have been better to have other people than MS and AH do the confidence assessments since they have already done the extraction. No use crying over spilt milk though.

Page 8 I think it might be better to move some of the detail here into a tabular format and just give the high–level numbers in the text. This would make it easier to see the wood for the forest. In fact I think much of the detail is already provided elsewhere.

Page 9 I did not find the list of limitations very convincing especially the ones about privacy, confidentiality and data security. I would expect to see that in detail when I review a grant application but not in the final publication.

Page 11 What does it mean for the quantitative studies to ‘extend’ as opposed ‘support’?

Page 21 In comparison with many published reviews I felt coverage of sub–Saharan Africa was quite good in terms of number of countries, certainly better than non–Anglophone America and anywhere east of the Oder/Neisse line.

Points of more substance

Mixed–methods

The authors refer to the issue of doing or reporting genuinely mixed–methods research. I feel they have done a good job here in presenting an integration of the qualitative and quantitative studies. I still have some doubts arising from presenting the qualitative as the major evidence and then the quantitative as informing it. Would it have been possible for the quantitative to flatly contradict the qualitative or present entirely novel themes?

The authors found a small number of mixed methods studies. I am not clear how these were incorporated into their narrative.

Trial recruitment in general

I am surprised that there does not seem to be any discussion here about whether these findings are consistent with other work on recruitment of women who are not pregnant or indeed people in general into trials. Although I have been involved in a number of trials over the years and have sat on trial oversight committees I am afraid I am quite ignorant of general literature about research into trial recruitment but I assume it must exist.

Summary

Mostly for clarification but a couple for more thought.

Michael Dewey

References

J L Jackson, A Kuriyama, A Anton, A Choi, J-P Fournier, A-K Geier, F Jacquerioz, D Kogan, and R Sun. The accuracy of Google Translate for abstracting data from non–English–language trials for systematic reviews. *Annals of Internal Medicine*, 171:677–679, 2019.
