# Supplementary material for: Factors influencing the participation of pregnant and lactating women in clinical trials: A mixed-methods systematic review
Source: PLoS Med. 2024 May 30;21(5):e1004405. doi: 10.1371/journal.pmed.1004405 (PMC11139290; doi:10.1371/journal.pmed.1004405)
Supplement: Attachment — Submitted filename: Response to reviewers_UpdatedPgNos_20240328.docx [file pmed.1004405.s009.docx]

15 March 2024

Dear PLOS Medicine Editorial Team,

We are grateful to the three reviewers for their feedback on our manuscript. Please find on Page 2 a table with each of the reviewers’ and editorial team’s comments, our responses, and, if applicable, the page and line numbers where changes have been made in the revised manuscript.

Sincerely,

Mridula Shankar (and on behalf of the manuscript’s co-authors)

| **Reviewer feedback** | **Authors’ response** |
| --- | --- |
| **REVIEWER 1** |  |
| Page 4. I can understand most of the exclusions but why exclude dissertations and theses? I suppose one could argue that high quality ones will have, at least in part, been published but I doubt that is universal. | We had initially planned to include a search of gray literature; however, our database searches yielded a high number of publications (n=27,867), Therefore, we made a pragmatic decision to exclude gray literature data. To address this comment, we have added this as a limitation of our systematic review in the discussion section (page 23, lines 980-982):  A limitation is that we did not include grey literature, which may have expanded the types of evidence and/or contexts of the review. However, our search strategies yielded high coverage of published literature. |
| Page 5. Why was substance use excluded? We ought to know the reason. | We excluded records concerning substance use therapy in pregnant women as the co-author team felt that this category of records would reveal particularly distinct barriers and facilitators given overlapping vulnerabilities among substance using pregnant women, and unique considerations in relation to fetal health such as in-utero exposure to alcohol and substances such as cannabis, opioids or other drugs. We have added this explanation in the manuscript as below (Page 6, lines 202-206):  (h) research solely focused on substance use prevention and treatment, due to the particularly distinct barriers and facilitators given overlapping vulnerabilities among substance-using pregnant women, and unique considerations in relation to fetal health such as in-utero exposure to alcohol and other substances. |
| Page 5. Good to see a specialist in information involved, a neglected source of expertise. | Thank you. We are indeed grateful for having access to this specific expertise within our review team. |
| Page 5. I am surprised that the authors used a generic AI system rather than a specialist machine translation service like DeepL or Google Translate.  There is some limited empirical work on using Google Translate in  reviews (Jackson et al., 2019). Having the outcome checked by a human is clearly a good idea.  References  J L Jackson, A Kuriyama, A Anton, A Choi, J-P Fournier, A-K Geier,  F Jacquerioz, D Kogan, and R Sun. The accuracy of Google Translate for abstracting data from non-English-language trials for systematic reviews. *Annals of Internal Medicine*, 171:677-679, 2019. | Thank you for the reference.  We did consider using Google Translate, but on comparing translated text generated from Google Translate and Chat GPT, we found the Chat GPT translations more legible, prompting our use of the latter system. To overcome concerns about sole reliance on AI generated translations, we cross-checked all abstracted data from the Chat GPT translated text with a native speaker colleague who used the original-language document as their point of reference. |
| Page 6. It might have been better to have other people than MS and AH do the confidence assessments since they have already done the extraction. No use crying over spilt milk though. | We note that the approach and methods we used of engagement of the review team (specifically MS, AH, MAB) in the screening, extraction, critical appraisal, analysis, and GRADE-CERQual confidence assessments is the standard approach for these types of reviews, and recommended by the GRADE-CERQual methods team (of which MAB is co-lead). See for example:   1. GRADE-CERQual methods paper: <https://implementationscience.biomedcentral.com/articles/10.1186/s13012-017-0689-2> 2. Cochrane template for conducting qualitative evidence syntheses: <https://zenodo.org/records/5973704> |
| Page 8. I think it might be better to move some of the detail here into a tabular format and just give the high level numbers in the text. This would make it easier to see the wood for the forest. In fact I think much of the detail is already provided elsewhere. | Thank you for this suggestion. We have included a new table 1 that provides percentage distributions of a range of characteristics of the included studies. We have substantially reduced the amount of detail provided in the main-text. |
| Page 9. I did not find the list of limitations very convincing especially the ones about privacy, confidentiality and data security. I would expect to see that in detail when I review a grant application but not in the final publication. | We used an **adapted** version of the 2018 Mixed Methods Appraisal Tool (MMAT) designed for use in the quality appraisal records included in mixed methods systematic reviews (link below). We chose this tool as it permits the appraisal of the methodological quality of quantitative, qualitative and mixed methods empirical studies, alleviating the need to use separate tools for each design.  The adapted version of the tool includes the dimension in question, namely whether ethical issues such as consent, confidentiality, data security etc were taken into consideration. We included this dimension as increasingly, standards for reporting research ask for these details to be included in manuscripts.  As approaches and criteria to evaluate quality of included studies evolve over time, we also understand that there are likely to be differences in opinion regarding the value of specific appraisal criteria. To enhance transparency in this process, we offer a detailed presentation of the ratings for each criterion in table X, and importantly, we do not exclude studies from the review judged by the review team to be of low methodological quality.  Link to the MMAT tool: <http://mixedmethodsappraisaltoolpublic.pbworks.com/w/file/fetch/127916259/MMAT__criteria-manual_2018-08-01_ENG.pdf> |
| Page 11 What does it mean for the quantitative studies to `extend' as opposed `support'? | Thank you for this query. In mapping the findings of the quantitative studies onto the findings of the qualitative evidence synthesis (QES), we first determined whether the quantitative findings reported information that was similar or identical to the QES finding. For example, QES finding #8 regarding pregnant women choosing to participate in clinical research due to altruistic motivations was supported by findings of quantitative studies that similarly reported altruism as a contributory factor. The quantitative studies that discussed altruism did not add any specific details regarding this motivating factor that hadn’t already been discussed in studies contributing to the QES. We therefore regarded the quantitative data as “supporting” our QES findings.  In the case of other findings we found that there were details in the findings of quantitative studies that were previously not evidenced in the qualitative studies contributing to a QES finding. This was the case for QES finding #9 on financial incentives, where the quantitative data extended our QES finding that pregnant and lactating women had mixed attitudes to financial incentives, by suggesting that attitudes to financial compensation differed based on levels of education attainment. In our view, the quantitative studies contributed additional details to the QES finding by indicating a variable on the basis of which attitudes to financial compensation may differ.  We have added the following details in the “Data analysis and synthesis” section of the manuscript on page 8, lines 286-290, to clarify our approach to mapping of the quantitative data on the QES findings.  We regarded the quantitative data as (a) “supporting” of a qualitative evidence synthesis finding if the information synthesised from the contributory quantitative studies was similar to the finding; (b) “extending” if the data offered additional details in line with a review finding, and (c) “contradictory” if the data conflicted with a review finding. |
| Page 21 In comparison with many published reviews I felt coverage of sub-Saharan Africa was quite good in terms of number of countries, certainly better than non-Anglophone America and anywhere east of the Oder/Neisse line. | Thank you for this observation with which we agree. We have revised the paragraph in the Discussion section addressing the geographical distribution of included records on page 23, lines 982-995 as follows:  The studies included in the review had good coverage of countries from the African region, but sparse representation of countries from Latin America, and no representation of countries in the Eastern Mediterranean or South-East Asian regions. A growing number of trials addressing maternal and perinatal health are being implemented in these settings [106], calling for significantly greater focus in formative and process evaluation research with women, family members, health workers, local researchers and ethics committee members to understand context-specific motivations for and concerns regarding conduct of and participation in research during pregnancy and lactation. |
| Mixed methods  The authors refer to the issue of doing or reporting genuinely mixed methods research. I feel they have done a good job here in presenting an integration of the qualitative and quantitative studies. I still have some doubts arising from presenting the qualitative as the major evidence and then the quantitative as informing it. Would it have been possible for the quantitative to contradict the qualitative or present entirely novel themes? The authors found a small number of mixed methods studies. I am not clear how these were incorporated into their narrative. | Thank you for raising this important point. In conducting a mixed methods systematic review, our decision to start with presenting the qualitative evidence and conduct a qualitative evidence synthesis to develop the review findings was based on two key reasons: 1) the richness of evidence from qualitative research, and 2) the data contributing to the review was disproportionately weighted towards qualitative studies (39 qualitative studies versus 18 quantitative studies). We anticipated this to be the case, given the nature of evidence that we were looking for, regarding factors affecting clinical trial engagement, which are often captured as part of qualitative process evaluations of randomized clinical trials (RCTs). Furthermore the quantitative studies contributing to this review are not randomised controlled trials, but rather descriptive observational studies.  Methodological guidance on conducting qualitative evidence syntheses have increased over the past decade, and our review team was of the opinion that our review would be conceptually richer and thicker in description if we used a QES approach as a basis for synthesis of the data.  Presently, we are unaware of methodological guidance that has sought to genuinely integrate at the point of analysis diverse forms of evidence from data sources that differ in their epistemological stances. Often mixed methods reviews will conduct a meta analysis of RCTs to generate a synthesis on effectiveness, alongside a qualitative evidence synthesis of patient experience. However, in these instances, the quantitative data sources are still evaluated using different methods, with integration taking place in the discussion or explanation of findings. Our mixed methods review is not conducive to such an approach. Instead, we use the framework of deriving review findings from the qualitative evidence synthesis (for reasons mentioned previously) and subsequently mapping the quantitative data on to review findings, and assessing whether the quantitative data supports, extends or contradicts the review finding.  Our review included three mixed methods papers. Qualitative data from these papers contributed to the source data for the qualitative evidence synthesis, and quantitative data from the papers contributed source data to the quantitative findings that were mapped to review findings. |
| Trial recruitment in general  I am surprised that there does not seem to be any discussion here about whether these findings are consistent with other work on recruitment of women who are not pregnant or indeed people in general into trials. Although I have been involved in a number of trials over the years and have sat on trial oversight committees I am afraid I am quite ignorant of general literature about research into trial recruitment but I assume it must exist. | Thank you for raising this observation. We have integrated findings of reviews and studies in other areas (e.g., participation in cancer trials) regarding factors affecting clinical trial enrolment in the Discussion on Page 21, lines 914-919 as follows:  Some of these findings, such as the role of altruism and potential for personal benefit, concerns about randomisation and other study design features, burdensome trial procedures, fears associated with taking an experimental therapy, and health worker attitudes towards trials are also consistent with the broader literature on factors associated with trial participation [99-101]. |
| **REVIEWER 2** |  |
| In this manuscript, the authors present a sweeping and comprehensive systematic review of qualitative and quantitative studies addressing the participation of pregnant and lactating women in clinical trials. This mixed methods review is very well done and contains excellent information. A few comments and questions follow. | Thank you. |
| 1. The introduction is excellent and provides a sound basis for the study. This could be an introduction given to policymakers and stakeholders in its current form. | Thank you very much. |
| 2. Page 5 first paragraph listing the exclusion on letter F-why were studies excluded if they looked at engagement in observational research? Given that the entire focus of the paper was to analyze and synthesize facilitators and barriers to engaging pregnant women in research, how is it justifiable to exclude inclusion in observational studies? | Thank you for raising this important question. This review focused specifically on factors affecting engagement of pregnant and lactating women in interventional clinical research. We restricted this review to participation in interventional research due to documented historic and ongoing exclusions of these populations from pharmaceutical and vaccine research which has resulted in pregnant and lactating women not adequately benefitting from innovations in biomedical research. Such under-representation and the associated health implications were widely documented in the context of the COVID-19 pandemic, and regional Zika and Ebola epidemics in South America and West Africa respectively where despite higher risk of severe illness and mortality for the maternal-fetal dyad, pregnant women were systematically excluded from early vaccines and investigational treatments without adequate scientific justification.  Notwithstanding the importance of observational research with pregnant and lactating women, and related factors associated with participation, we believe that participation in interventional research brings with it a unique set of facilitators and barriers, including (but not limited to) risk-benefit assessments for the maternal-fetal dyad, that affect all levels of the research enterprise from research funding at one end to engaging health workers to administer and individuals to participate in clinical research trials, at the other end. |
| 3. The findings overall were things that have been talked about for years. One of the key things that was exciting about this manuscript was the potential to put together a list of accomplishable behavior change strategies. However, the manuscript does not really do this in an effective way. Table 2 maps the findings to behavior change interventions, but still has 24 different things to accomplish. This becomes overwhelming and demotivating for investigators, participants, and governing and regulatory bodies. It would have been extremely helpful for a shorter prioritized list to be developed for change strategies that could be implemented quickly. | Thank you for this observation. Our goal in mapping findings to behavior change interventions was to provide a theoretically informed overview of the types of strategies that can be employed to address a range of barriers targeting various stakeholders. However, these strategies are not intended to be universally applicable or of equal priority across every context and setting. Instead, we believe that the choice of which strategies to apply, and how they must be adapted are decisions that should be made by stakeholders who have context-specific knowledge and expertise. We have added text to this section on Page 20, lines 853-857, indicating as such:  The strategies that we have identified are designed to provide a theoretically informed guide to the types of actions that can be taken to address barriers at various levels associated with different stakeholder groups. Which actions are appropriate for a given context should therefore be discussed, prioritised, and adapted to a particular setting. |
| 4. Many of the 24 different findings are somewhat related and could have been collapsed together. | Thank you for noting this observation. We agree that many of the review findings are related. This is not unexpected as individual factors affecting participation rarely operate in isolation, and often co-occur or have linkages with other factors and stakeholders. Our approach to synthesising the data involved determining, discussing and identifying conceptual boundaries between findings, collapsing findings into common conceptual domains, and retaining review findings as separate when boundaries were distinct (even if concepts were related), including when contributory studies to each review finding differed. Given our systematic approach to developing review findings using a qualitative evidence synthesis methodology and our use of the GRADE-CERQual approach to assessing level of confidence in individual review findings, we have made no revisions to the set of qualitative findings. We note that the number of findings is appropriate and aligned with other recently published reviews using similar approaches:   1. Cochrane qualitative evidence synthesis on postpartum hemorrhage: <https://www.cochranelibrary.com/cdsr/doi/10.1002/14651858.CD013795.pub2/full> 2. Mixed-methods review on antenatal corticosteroids: <https://journals.plos.org/plosmedicine/article?id=10.1371/journal.pmed.1004074> |
| **REVIEWER 3** |  |
| I have a read the manuscript with great interest. As the authors say, their systematic review is an elaboration of a previous systematic review performed by Van der Zande et al (2018) that looked at women's reasons for participation in clinical research. Since the authors took a more comprehensive approach (a broader group of stakeholders and a broader set of studies), the result is impressive: 24 well-considered key findings. | Thank you. |
| A well-known problem is that studies that measure the willingness to participate are often based on retrospective data and lead to recall bias. Could data be added about the type of studies that was found? | Thank you for this observation. Study designs which rely on participants needing to recall past events are exposed to recall bias when there are differential rates of recall between cases and controls, or if the duration of time between “events” and data collection affect the ability for people to recall the events themselves or items related to the event.  Our included records were a mix of (a) qualitative and quantitative studies that sought to understand the reasons why individuals chose to participate or decline specific trials, (b) qualitative studies interviewing researchers, ethics committee members, funders etc., about the motivations and challenges in including pregnant and lactating women in clinical trials, (c) qualitative research with women, family and community members about attitudes to trial participation, (d) surveys with providers and providers-in-training about their attitudes towards pregnant women participating in trials, and (e) scenario-based surveys and qualitative interviews regarding participation in hypothetical vaccine trials.  In studies where participants were asked to recall their reasons for participating or declining participation in a clinical trial, this information was obtained within a relatively short time of the decision made; hence the possibility of participants recalling incorrectly or failing to recall information relevant to the research question is unlikely to be a major concern in these studies. |
| Could data be added about the type of clinical research that was studied? As the authors acknowledge in the introduction there is a scarcity on pregnancy specific medications. Can more be said on the type of studies found? Obstetric, non-obstetric, pharmacological, non-pharmacological? | Thank you for raising this question. We have added a new Table 1 that provides descriptive characteristics of the included studies, including information pertaining to the trial areas. Additionally, in the table S6.Appendix: Characteristics of included papers, we have included relevant data in two columns: (a) type of intervention (pharmaceutical therapy/vaccine/non specified), and (b) Trial area (complications of pregnancy/childbirth, infectious disease, non-communicable disease, fetal/newborn health etc.,) |
| **EDITORIAL COMMENTS** |  |
| 1. The editorial team are in agreement with the reviewers that your manuscript was very considerately designed and reported and that it could make a valuable contribution to the field. | Thank you. |
| 2. We thank you for reporting your study in accordance with PRISMA guidelines and for including the checklist as supporting information. PLOS Medicine requires that systematic reviews are updated to within 6 months of an anticipated publication date. We appreciate the complexity of your particular study but please consider this point as part of your revision. | We have re-run all database searches to include records published up to 14 Feb 2024, and included four new records, two of which are qualitative studies (Dorey 2023 and Sweet 2023), and two are quantitative studies (Bevan 2023 and Scott 2023). Together, these records contribute data to 16 of the 24 review findings. The manuscript main-text, tables, figures and appendices have been updated accordingly. |
