# Supplementary material for: Factors influencing the participation of pregnant and lactating women in clinical trials: A mixed-methods systematic review
Source: PLoS Med. 2024 May 30;21(5):e1004405. doi: 10.1371/journal.pmed.1004405 (PMC11139290; doi:10.1371/journal.pmed.1004405)
Supplement: Attachment — Submitted filename: Response to reviewers.docx [file pmed.1004405.s010.docx]

**Response to reviewers’ and editorial team comments**

| **Reviewer feedback** | **Authors’ response** |
| --- | --- |
| **REVIEWER 1** |  |
| Reviewer #1: The authors have answered my queries and addressed my other points. | Thank you |
| **REVIEWER 3** |  |
| Thanks for addressing the comments. I had asked for table 1 and table S6, we I could not find in the original resubmission. The additions in table 1 are helpful. And table S6 appears to be a typo (it should be Appendix S6). If these small comments are addressed than I have no further questions. | Thank you. We have checked S6 Appendix but are unable to locate the typo found by Reviewer 3. |

| **EDITORIAL COMMENTS** |  |
| --- | --- |
| 1. ABSTRACT   In the last sentence of the Abstract Methods and Findings section, please (briefly) describe the main limitation(s) of the study's methodology. | We have now included a key limitation in the relevant section in the abstract as below (page 2, lines 67-70 in the track changes manuscript):  All included studies focused on factors affecting participation of cisgender pregnant women in clinical trials; future research should consider other pregnancy-capable populations, including transgender and non-binary people. |
| 2. AUTHOR SUMMARY   Thank you for including an author summary which reads very nicely. In the final bullet point of ‘What Do These Findings Mean?’, please describe the main limitations of the study in non-technical language. We appreciated the inclusive reference to ‘lactating women and people’ in the opening bullet points but the same isn’t continued throughout. I noted your clarification regarding terminology Vs applicability to gender diverse populations (methods, line 182 onwards) which is very helpful to the reader and to me explains the aforementioned. Perhaps including a similar statement as part of the limitations (as requested above) would mitigate against any confusion regarding your use of terminology in the opening bullet points and the description of the research outcomes latterly. | Thank you for this helpful suggestion. We have now included a third bullet point under “What do these findings mean” which reads as follows:     - All included studies focused on factors affecting participation of cisgender pregnant women in clinical trials; future research should consider other pregnancy-capable populations, including transgender and non-binary people. |
| 3. INTRODUCTION   A As above, please consistently refer to ‘women and people’, (or populations) line 124, for example. Please check carefully throughout the complete manuscript (including the supporting information) and revise as necessary. | We have checked all aspects of the manuscript and associated tables and have revised accordingly. Please note that in the Introduction section:   1. We use the phrase “pregnant women and people” when referring to the research needs and interests of individuals who are pregnant. 2. Retained “pregnant women” when using empirical data where the original referenced source refers to cisgender women only. This would include, for example, line 140 on Page 4, as below, where the associated reference (#11), only uses “women” in the publication. |
| 4. TABLES   P Please ensure that all tables (including those in the supporting information) are affiliated to an appropriate title and caption which clearly describes their content without the need to refer to the text. | Tables have been checked and updated.  We have made the following changes to table titles:     1. Table 1.   Original title: “Characteristics of included studies (n=60)”.  Updated title: “Summary of characteristics of Included papers”  This change was made to distinguish the title of Table 1 from the title of S6 Appendix, and to distinguish between papers and studies, since we included 60 papers from 53 studies.     2. Table 3.   Original title: “Mapping of Review Findings to TDF and COM-B Domains and Behaviour Change Wheel Intervention Types”  Updated title: “Mapping qualitative findings to the TDF and COM-B frameworks and Behaviour Change Wheel”     3. S1 Appendix:   Original title: “Preferred Reporting Items for Systematic Reviews and Meta-Analyses (PRISMA) reporting guideline”  Updated title: “Preferred Reporting Items for Systematic Reviews and Meta-Analyses (PRISMA) reporting checklist”   4. S2 Appendix:   Original title: “Enhancing Transparency in Reporting the Synthesis of Qualitative Research: (ENTREQ) statement”  Updated title: “Enhancing Transparency in Reporting the Synthesis of Qualitative Research (ENTREQ) reporting checklist”     5. S6 Appendix:   Original title: “Characteristics of included studies”.  Updated title: Characteristics of Included Papers”     6. S7 Appendix:   Original title: “Results of assessment of methodological limitations of included studies”  Updated title: “Critical Appraisal” |
| 5. DISCUSSION   Pl Please present and organize the Discussion as follows: a short, clear summary of the article's findings; what the study adds to existing research and where and why the results may differ from previous research; strengths and limitations of the study; implications and next steps for research, clinical practice, and/or public policy; one-paragraph conclusion. | We have reviewed the Discussion and aside from making minor wording and formatting changes, we have retained the content as-is as we feel it meets the requirements specified here as below:   1. Paragraph 1, lines 878-891: summary of study findings 2. Paragraphs 2, 3, 4 and 5, lines 893-952: what the study adds and how findings compare with existing research evidence on the topic, and the implications for conduct of clinical research with pregnant populations 3. Paragraph 6, lines 954-959:  The value of COM-B and TDF mapping in our review and how it can be used to inform strategies to address barriers to research inclusion of pregnant and lactating populations. 4. Paragraph 7, lines 961-978: Limitations and strengths of this study 5. Paragraph 8, lines 980-994: Conclusion |
| 6. REFERENCES   For For in-text reference callouts please place citations in square parentheses separate by commas but no additional spaces. For example, [1,3,6] or [1-3]. Please check and amend throughout all sub-sections of the manuscript and supporting files.  In In the bibliography please ensure that you list up to but no more than 6 author names followed by et al.  For all web references please ensure you include an, ‘Accessed [date].’  Jou Journal name abbreviations should be those listed in the National Cen Center for Biotechnology Information (NCBI) databases.  SUPPORTING INFORMATION  SSu  Ple Please ensure to cite your Supporting Information as outlined here: <https://journals.plos.org/plosmedicine/s/supporting-information>  In In the published article, supporting information files are accessed only through a hyperlink attached to the captions. For this reason, you must list captions at the end of your manuscript file. You may include a caption within the supporting information file itself, as long as that caption is also provided in the manuscript file. Do not submit a separate caption file.  Pl Please ensure to upload a clean (as well a tracked) version of supporting information files. | References checked and updated. |
| 7. SOCIAL MEDIA   To help us extend the reach of your research, please detail any X (formerly Twitter) handles you wish to be included when we tweet this paper (including your own, your coauthors’, your institution, funder, or lab) in the manuscript submission form when you re-submit the manuscript.  To | Mridula Shankar: @mr1dula  Alya Hazfiarini: @ahazfiarini  Rana I. Zahroh: @ranazahroh  Joshua P. Vogel: @josh_vogel  Annie R.A. McDougall: @DrAMcDougall  A. Metin Gülmezoglu: @gulmezoglum  Meghan A. Bohren: @meghanbohren |
|  | Miscellaneous changes:   1. In the Introduction section, we have made a minor modification on Page 4, line 141, removing the phrase “off-label”. This change has been made, as the term off-label may not be easily interpretable by the reader, and the sentence does not lose any meaning with the phrase removed. |
